# Supplementary material for: Targeting natural splicing plasticity of APOBEC3B restricts its expression and mutagenic activity
Source: Commun Biol. 2021 Mar 22;4:386. doi: 10.1038/s42003-021-01844-5 (PMC7985488; doi:10.1038/s42003-021-01844-5)
Supplement: Supplementary file 1 — Supplementary Information [file 42003_2021_1844_MOESM1_ESM.pdf]

Supplementary Materials

Targeting natural splicing plasticity of APOBEC3B restricts its expression and mutagenic activity

A. Rouf Banday[1], Olusegun O. Onabajo[1], Seraph Han-Yin Lin[1], Adeola Obajemu[1], Joselin M. Vargas[1], Krista A. Delviks-Frankenberry[2], Philippe Lamy[3], Ariunaa Bayanjargal[1], Clara Zettelmeyer[1], Oscar Florez-Vargas[1], Vinay K. Pathak[2], Lars Dyrskjøt[3], Ludmila Prokunina-Olsson[1*]

[1]Laboratory of Translational Genomics, Division of Cancer Epidemiology and Genetics, National Cancer Institute, National Institutes of Health, Bethesda, MD, USA

[2] Viral Mutation Section, HIV Dynamics and Replication Program, Center for Cancer Research, National Cancer Institute, Frederick, MD, USA

[3]Department of Molecular Medicine, Aarhus University Hospital, Aarhus, Denmark

Section details	Title	Page
**Tables**		
Supplementary Table 1	Isoforms of the *APOBEC3* genes analyzed in TCGA samples	3
Supplementary Table 2	TCGA samples used in the analysis	4
Supplementary Table 3	Analysis of splicing factor binding sites within exons of A3B 5-6 isoforms affected by alternative splicing	5 – 6
Supplementary Table 4	Expression constructs for alternative splicing factors used for 7 co-expression studies	7
Supplementary Table 5	Summary of Human Splicing Finder analysis of branch point 8 sites within *A3B* introns	8
**Figures**		
Supplementary Fig. 1	Splicing patterns of A3A and A3B genes in TCGA tumor and adjacent normal tissues (N=11,058) based on RNA-seq read	9
Supplementary Fig. 2	Evaluation of possible dominant-negative effects of catalytically inactive A3A and A3B isoforms	10
Supplementary Fig. 3	Functional evaluation of A3B protein isoforms using HIV-1 infectivity inhibition assays	11
Supplementary Fig. 4	Distribution of APOBEC-signature mutations in TCGA tumors	12
Supplementary Fig. 5	Percent spliced-in index (PSI) for alternative splicing isoforms of *A3B* in matched paired tumor and adjacent normal tissues in TCGA	13
Supplementary Fig. 6	Analysis of alternative splicing in A3A and A3B with Exontrap mini-gene assays	14
Supplementary Fig. 7	Overexpression of SRSF2 increases A3B exon 5 skipping in cancer cell lines	15
Supplementary Fig. 8	Effects of pladienolide B treatment and siRNA-*SF3B1* knockdown on A3B exon 5 skipping in cancer cell lines	16
Supplementary Fig. 9	Cell-based cytosine deamination assay	17
Supplementary Fig. 10	Analysis of APOBEC-mediated mutagenesis in six TCGA cancer types in relation to mutations in select splicing factors	18
Supplementary Fig. 11	Unprocessed blots and gels	19 – 26
**Notes**		
Supplementary Note 1	Analysis of A3A and A3B transcripts based on exon-exon junctions and total expression using qRT-PCR and RNA-seq	27 – 30
Supplementary Note 2	Evaluation of the available antibodies for the detection of A3A and A3B protein isoforms	31 – 32
Supplementary Note 3	Purification/enrichment of recombinant A3A and A3B protein isoforms	32
Supplementary Note 4	Isoform-specific TaqMan expression assays for A3B transcripts	33
Supplementary Note 5	Correlation analysis between A3B exon 5 skipping and mRNA expression of select splicing factors in cell lines	34
**Supplementary References**		35

TABLES

Supplementary Table 1. Isoforms of the *APOBEC3* genes analyzed in TCGA samples

Gene	Isoform ID in TCGA RNA-seq	UCSC ID in hg19	Designation in the paper	Isoform type	Splicing event
A3A	uc003awn.2	uc003awn.2	A3A1	Canonical	N/A
A3A	uc011aob.1	uc011aob.1	A3A2	Alternative	Cryptic splice site in exon 2
A3AB	uc011aoc.1	uc011aoc.1	A3AB	Deletion	N/A
A3B	uc003awo.1	uc003awo.2	A3B1	Canonical	N/A
A3B	uc003awp.1	uc003awp.2	A3B2	Alternative	Cryptic splice site in exon 6
A3B	uc003awq.1	uc003awq.2	A3B3	Alternative	Skipping of exon 5

Supplementary Table 2. TCGA samples used in the analysis

Number	Abbreviation	Cancer type	Samples, N#
1	ACC	Adrenocortical Carcinoma	92
2	BLCA	Bladder Urothelial Carcinoma	412
3	BRCA	Breast Invasive Carcinoma	1098
4	CESC	Cervical Squamous Cell Carcinoma and Endocervical Adenocarcinoma	307
5	CHOL	Cholangiocarcinoma	51
6	COAD	Colon Adenocarcinoma	460
7	DLBC	Lymphoid Neoplasm Diffuse Large B-cell Lymphoma	58
8	ESCA	Esophageal Carcinoma	185
9	GBM	Glioblastoma Multiforme	613
10	HNSC	Head and Neck Squamous Cell Carcinoma	528
11	KICH	Kidney Chromophobe	113
12	KIRC	Kidney Renal Clear Cell Carcinoma	537
13	KIRP	Kidney Renal Papillary Cell Carcinoma	323
14	LAML	Acute Myeloid Leukemia	200
15	LGG	Brain Lower Grade Glioma	516
16	LIHC	Liver Hepatocellular Carcinoma	377
17	LUAD	Lung Adenocarcinoma	585
18	LUSC	Lung Squamous Cell Carcinoma	504
19	MESO	Mesothelioma	87
20	OV	Ovarian Serous Cystadenocarcinoma	602
21	PAAD	Pancreatic Adenocarcinoma	185
22	PCPG	Pheochromocytoma and Paraganglioma	179
23	PRAD	Prostate Adenocarcinoma	499
24	READ	Rectum Adenocarcinoma	171
25	SARC	Sarcoma	261
26	SKCM	Skin Cutaneous Melanoma	470
27	STAD	Stomach Adenocarcinoma	443
28	TGCT	Testicular Germ Cell Tumors	150
29	THCA	Thyroid Adenocarcinoma	503
30	THYM	Thymoma	124
31	UCEC	Uterine Corpus Endometrioid Carcinoma	560
32	UCS	Uterine Carcinosarcoma	57
33	OVM	Uveal Melanoma	80
Total			11330

Numbers of samples used for specific analyses may vary based on the availability of data for RNA-seq, APOBEC-signature mutations[1], and other variables (age, sex, race, etc).

Supplementary Table 3. Analysis of splicing factor binding sites within exons of *A3B* isoforms affected by alternative splicing

Isoform	Type of alternative splicing	Splicing event	The exonic sequence for SFs prediction, bp	SF	Another name	Target site, bp position within query sequence
**A3B2**	Between exon 5 and cryptic acceptor splice site in exon 6	Loss of 75 bp in exon 6	1-ggctaagaatcttctctgtggcttttacggccgccatgcggagctgcgcttcttggacctggttccttctttgca-75	CUG-BP	CELF1	UGCGG (37-41), AGCUG (42-46)
				HuB	ELAVL2	CUUUUA (22-27)
				hnRNP A1	HNRNPA1	UACGGC (26-31)
				hnRNPF	HNRNPF	GUGGCUU (18-24)
				hnRNP H1	HNRNPH1	AAGAA (5-9)
				hnRNP H2	HNRNPH2	AUCUUC (5-9)
				hnRNPM	HNRNPM	GGUUCCUU (61-68)
				Sam68	KHDRBS1	UUUUAC (23-28)
				MBNL1	MBNL1	GGCUUU (20-25, 46-51)
				hnRNP I (PTB)	PTBP1	AUCUUC (10-14), UCUUCUCU (10-17), UUCUUG (50-55), UUCUUU (67-72), UCUUU (68-72)
				SC35	SRSF2	GGCCGCCA (29-36), GGUUCCUU (61-68)
				SRp40	SRSF5	ACGGC (27-31), GCUGC (43-47)
				SRp55	SRSF6	UGCGGA (37-42)
				SRp30c	SRSF9	CGGAG (39-43), UGGAC (54-58)
				TIA-1	TIA1	CUUUUA (22-27), CUUUG (69-73)
				TIAL1	TIAL1	CUUUUA (22-27), CUUUG (69-73)
				HTra2alpha	TRA2A	AAGAA (5-9)
				HTra2beta1	TRA2B	AAGAA (5-9)
**A3B3**	Between exon 4 and exon 6	Loss of entire exon 5, 129 bp	1-gatacctgatggatccagacacattcactttcaacttgatacctgatggatccagacacattcactttcaacttcctggacaatggcacctgggtcctgatggaccagcacatgggctttctatgcaac-129	CUG-BP	CELF1	UGAUG (44-48), UCCUG (74-78), UCCUG (95-99)
				HuB	ELAVL2	CUUUC (28-32), CUUUC (65-69), CUUUC (117-121)
				hnRNP F	HNRNPF	UGGGC (113-117)
				hnRNP H1	HNRNPH1	UGGGU (91-95), UGGGC (113-117)
				hnRNP H2	HNRNPH2	UGGGU (91-95), UGGGC (113-117)
				hnRNP H3	HNRNPH3	UGGGU (91-95), UGGGC (113-117)
				KSRP	KHSRP	UGGGU (91-95)
				MBNL1	MBNL1	GGCUUU (115-120)

				Nova-1	NOVA1	UUCAAC (30-35), UUCAAC (67-72)
				Nova-2	NOVA2	UUCAAC (30-35), UUCAAC (67-72)
				SC35	SRSF2	UCAAC (31-35), UCAAC (68-72), ACUUC (71)
				SRp20	SRSF3	GAUC (12-15), UUCAC (24-28), GAUC (49-52), UUCAC (61-65)
				SRp40	SRSF5	UUCCUGG (73-79)
				9G8	SRSF7	UGGACAA (77-83)
				SRp30c	SRSF9	UGGAU (10-14), UGGAU (47-51), UGGAC (77-81), UGGAC (101-105)
				TIA-1	TIA1	CUUUC (28-32), CUUUC (65-69), CUUUC (117-121)
				TIAL1	TIAL1	CUUUC (28-32), CUUUC (65-69), CUUUC (117-121)
				HTra2beta1	TRA2B	UCAAC (31-35), UCAAC (68-72)
				YB-1	YBX1	GAUC (12-15), GAUC (49-52), CACC (87-90)

SF – splicing factor; bioinformatic predictions were performed with SFmap[2] and SpliceAid 2 (ref [3])

Supplementary Table 4. Expression constructs for alternative splicing factors used for co-expression studies

Construct	OriGene* catalog ID	Construct	NCBI ID	Matching UCSC Isoforms, hg19
1	RC206275	CELF1-T1	NM_006560	uc001nfn.3
2	RC222434	CELF1-T3	NM_001025596	uc001nfp.3, uc001nfl.3, uc001nfr.1
3	RC230208	CELF1-T4	NM_001172639	uc001nfk.2
4	RC229918	ELAV2	NM_001171195	uc003zpt.3
5	RC223971	TIAL1	NM_003252	uc001lei.1
6	RC200263	KHDRBS1	NM_006559	uc001buc.2, uc001bub.4
7	RC209660	SFRS3 (SRSF3)	NM_003017	uc003omj.3, uc011dtp.1
8	RC209842	SFRS2 (SRSF2)	NM_003016	uc002jsv.3, uc010wtg.2
9	RC203314	HNRNPA1	NM_002136	uc001sfo.3, uc009znj.1, uc001sfm.3, uc001sfn.3
10	RC219704	MBNL1	NM_207295	uc003ezn.3

*expression constructs were purchased from OriGene

Supplementary Table 5. Summary of Human Splicing Finder analysis of branch point sites within *A3B* introns

Intron of *A3B*	Position of the strongest motif, bp upstream of exon	BPS motif	Consensus score (0-100) for a top BPS in each intron	Number of potential BPS with a score above threshold (>67) as per HSF
1	-58	ccccgAg	83.31	3
2	-37	ctctcAg	93.91	6
3	-30	gcctgAc	93.44	3
4	-38	gtcttAg	80.45	2
5	-26	acctcAc	95.75	10
6	-77	tcctgAg	90.81	6
7	-40	ctctcAc	96.63	7

HSF - Human Splicing Finder www.umd.be/HSF3/; BPS – branch point site, represented by the upper case A within putative consensus motifs.

FIGURES

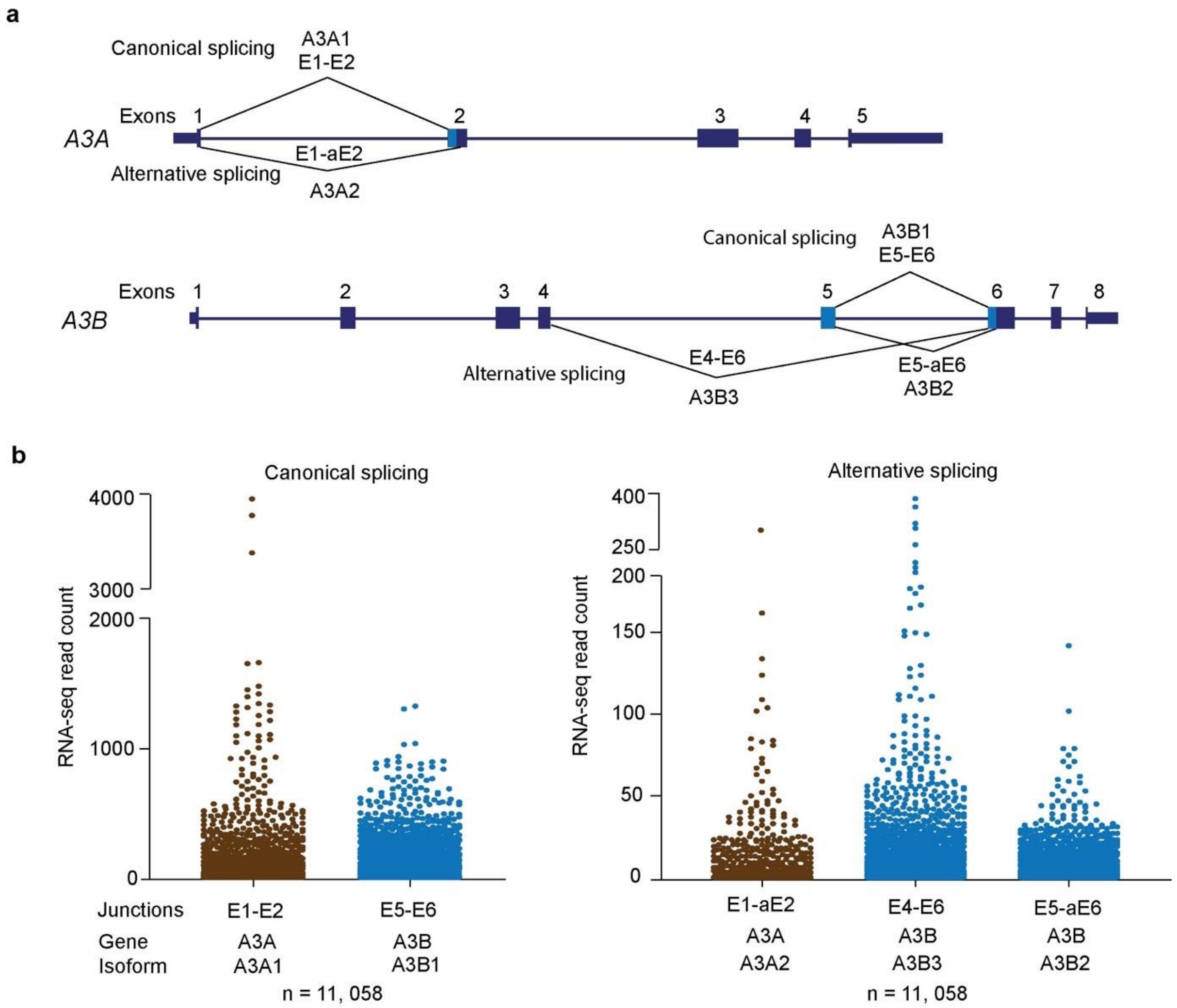

Supplementary Fig. 1. Splicing patterns of *A3A* and *A3B* genes in TCGA tumor and adjacent normal tissues (N=11,058) based on RNA-seq read counts for specific exon-exon junctions.

a) Two alternatively spliced isoforms were observed for *A3A* - the canonical isoform A3A1 defined by E1-E2 junction, and the alternative A3A2 isoform defined by E1-aE2 junction through a cryptic acceptor site in exon 2 (aE2). Three alternatively spliced isoforms were observed for *A3B* - the canonical isoform A3B1 defined by E5-E6 junction, and the alternative isoforms A3B2 and A3B3 defined by the usage of an alternative acceptor site (E5-aE6 junction), and exon 5 skipping (E4-E6 junction), respectively. **b**) Left panel: counts of RNA-seq reads supporting the canonical exon junctions E1-E2 and E5-E6 for A3A1 and A3B1 isoforms, respectively. Right panel: counts of RNA-seq reads supporting the alternative exon junctions E1-aE2 for A3A2, E5-aE6 for A3B2, and E4-E6 for A3B3.

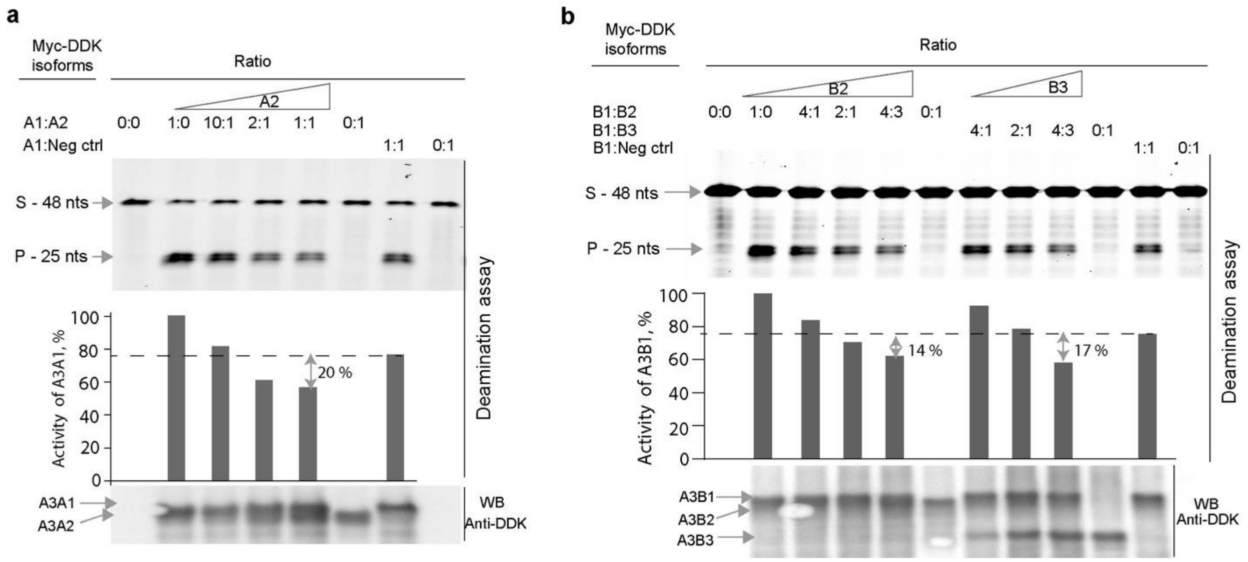

Supplementary Fig. 2. Evaluation of possible dominant-negative effects of catalytically inactive A3A and A3B isoforms.

a) Deaminating activity of the mutagenic protein isoform A3A1 was moderately inhibited (by 20%) by the corresponding non-mutagenic A3A2 protein isoform. **b**) Deaminating activity of the mutagenic isoform A3B1 was moderately inhibited (by 14 and 17%) by the corresponding non-mutagenic A3B2 and A3B3 protein isoforms, respectively. All assays were performed using enriched fractions of semi-purified proteins (**Note S3**). The amounts of mutagenic isoforms and total reaction volumes were kept constant while the amounts of non-mutagenic isoforms were increased. The deamination activity was compared to the mutagenic isoforms alone and to the mutagenic isoforms mixed in a 1:1 ratio with protein extracted from lysates of untransfected cells to account for inhibition caused by non-specific endogenous proteins. Plots show representative results of four independent experiments.

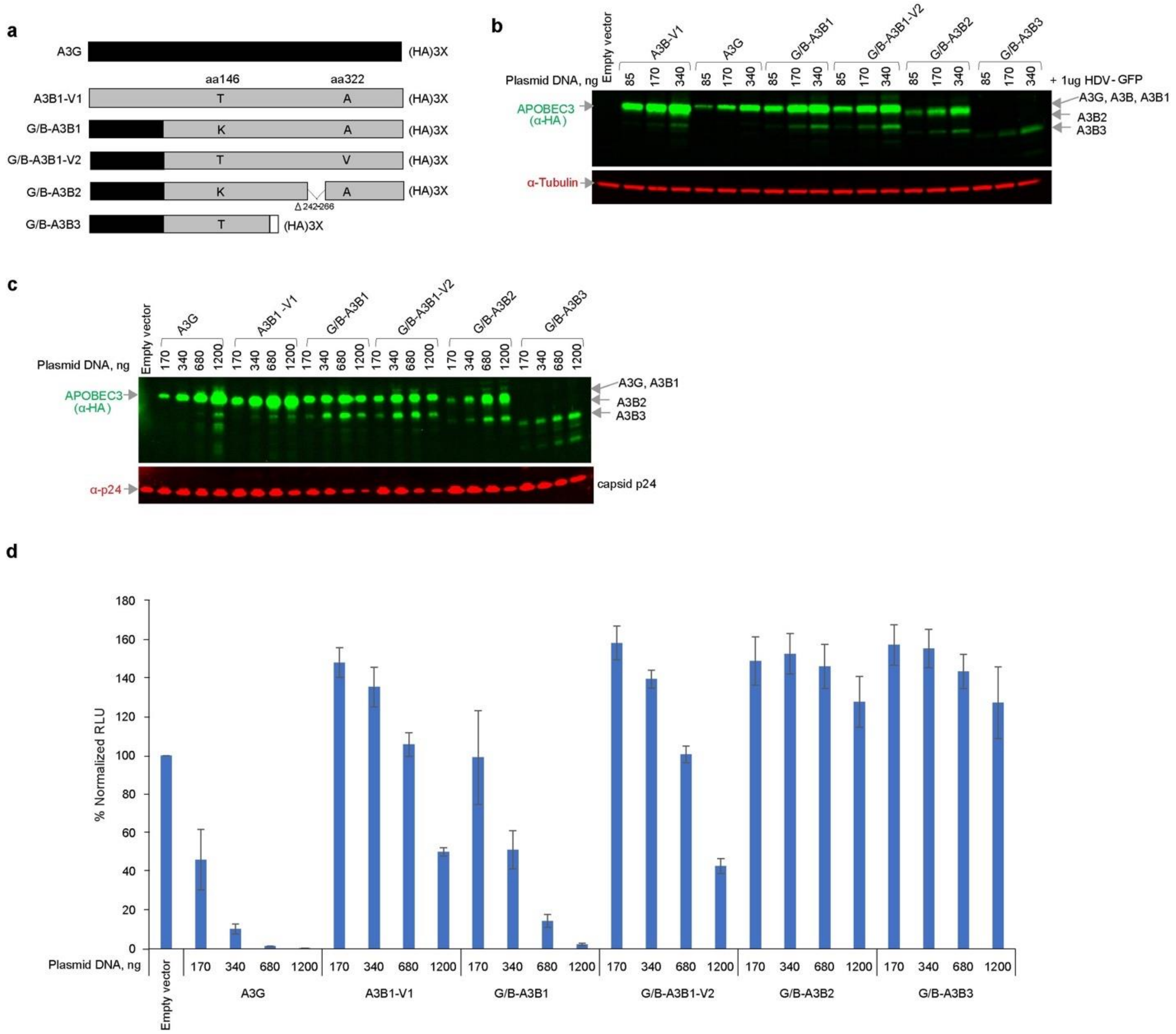

Supplementary Fig. 3. Functional evaluation of A3B protein isoforms using HIV-1 infectivity inhibition assays.

a) Fusion constructs were generated by replacing 63 aa of the N-terminal region of A3B with a similar region of APOBEC3G (A3G). **b**) All G/B fusion constructs were transfected into HEK293T cells; protein expression and stability were established as G/B-A3B1 = G/B-A3B1-V2 > G/B-A3B2 >>> G/B-A3B3. **c**) Western blotting is showing the viral packaging of recombinant proteins. Consistent with their steady-state expression levels, G/B-A3B2 and G/B-A3B3 fusions were incorporated into virions to a lesser extent than G/B-A3B1 and G/B-A3B2-V2. **d**) A3B2 and A3B3 proteins had no detectable effect on the inhibition of HIV-1 infectivity. As expected, based on previous reports[4,5], a fusion with the A3G N-terminal region enhanced the antiviral activity of A3B1 (compare A3B1-V1 with G/B-A3B1). A3B1-V1 inhibited HIV-1 infectivity in a dose-dependent manner, but A3B2 and A3B3 isoforms did not. All bar graphs show means ± SD of biological triplicates.

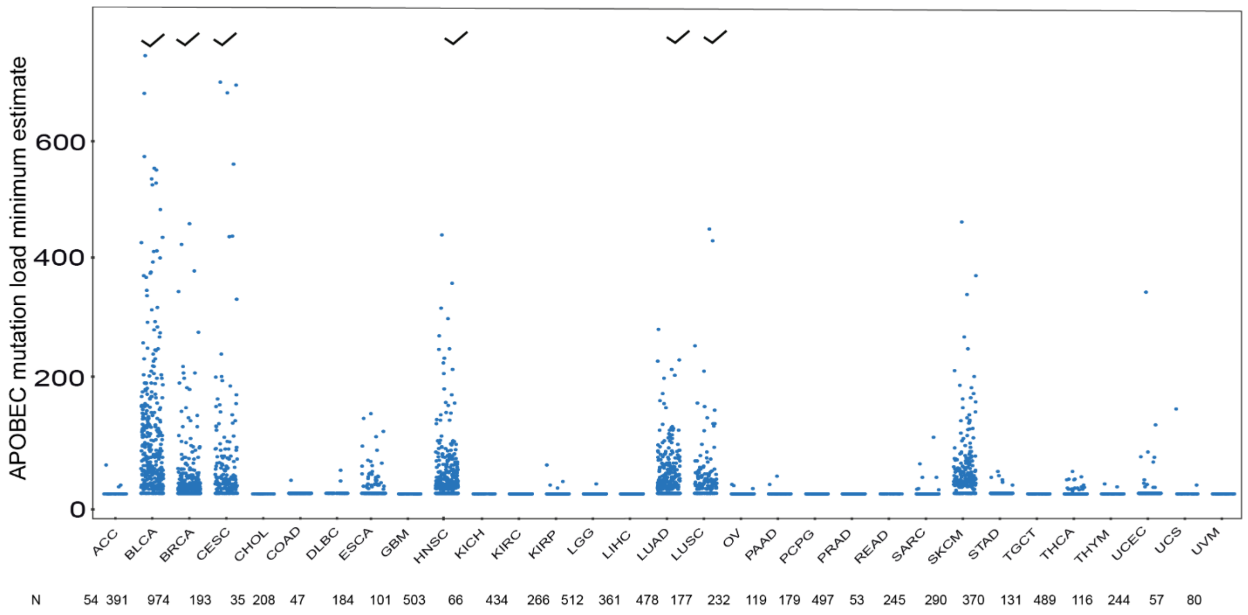

Supplementary Fig. 4. Distribution of APOBEC-signature mutations in TCGA tumors.

Abbreviations for cancer types are explained in **Supplementary Fig. 2**; each dot represents a tumor. N - the number of samples with mutations for each cancer type. Tick marks indicate cancer types with the highest numbers of tumors with APOBEC-signature mutations; a set of 6 cancer types (BLCA, BRCA, CESC, HNSC, LUAD, and LUSC) was selected for further analysis in relation to the expression of the mutagenic isoforms A3A1 and A3B1 (**Fig. 3**). Although SKCM is enriched in tumors with APOBEC-signature mutations, this cancer type was not analyzed because of the overlap between mutations caused by APOBECs and UV radiation[6].

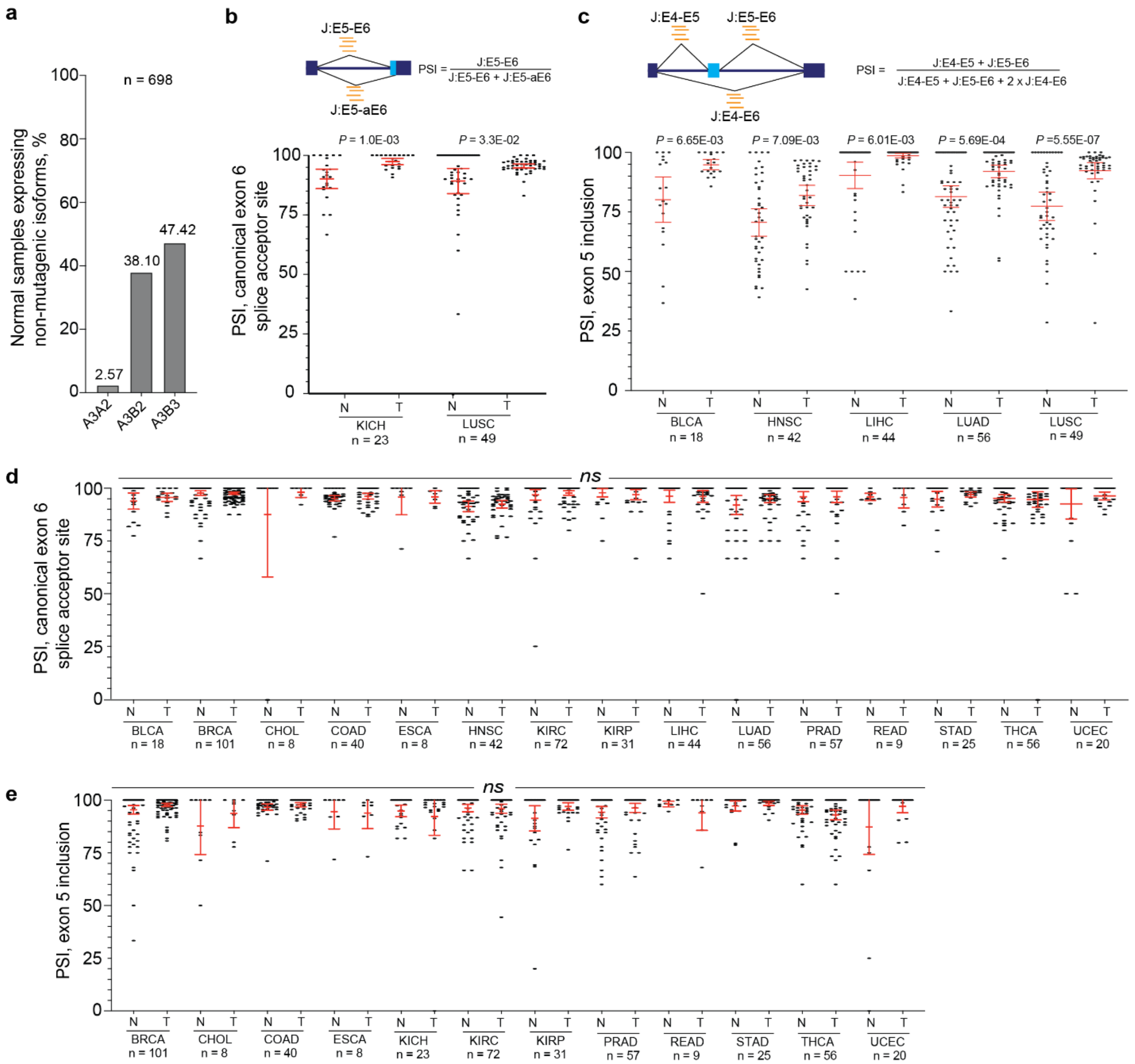

Supplementary Fig. 5. Percent spliced-in index (PSI) for alternative splicing isoforms of *A3B* in matched paired tumor and adjacent normal tissues in TCGA.

a) Percentage of matched normal samples expressing alternative isoforms of *A3A* and *A3B* with ≥1 RNA-seq reads for unique exon-exon junctions. **b** and **c)** The ratios of splicing events generating the canonical mutagenic A3B1 isoform **-** exon 5 inclusion and the use of canonical exon 6 acceptor site were calculated as percent spliced-in index (PSI) based on RNA-seq reads in TCGA. **b)** PSI values for the usage of canonical exon 6 acceptor site are significantly higher in tumors compared to normal samples for KICH and LUSC. **c)** PSI values for exon 5 inclusion are significantly higher in tumors (T) compared to normal (N) samples for BLCA, HNSC, LIHC, LUAD, and LUSC. **d** and **e**) PSI values for the canonical splicing isoforms - exon 5 inclusion and splicing through the canonical exon 6 acceptor site (E5-E6 junction) in several cancer types. These cancers show no significant differences (indicated as "*ns*") in PSI levels between matched paired tumor (T) and normal (N) samples. Dot plots show all individual data points and error bars represent 95% confidence intervals with means. *P*-values are for the paired non-parametric Wilcoxon signed-rank test.

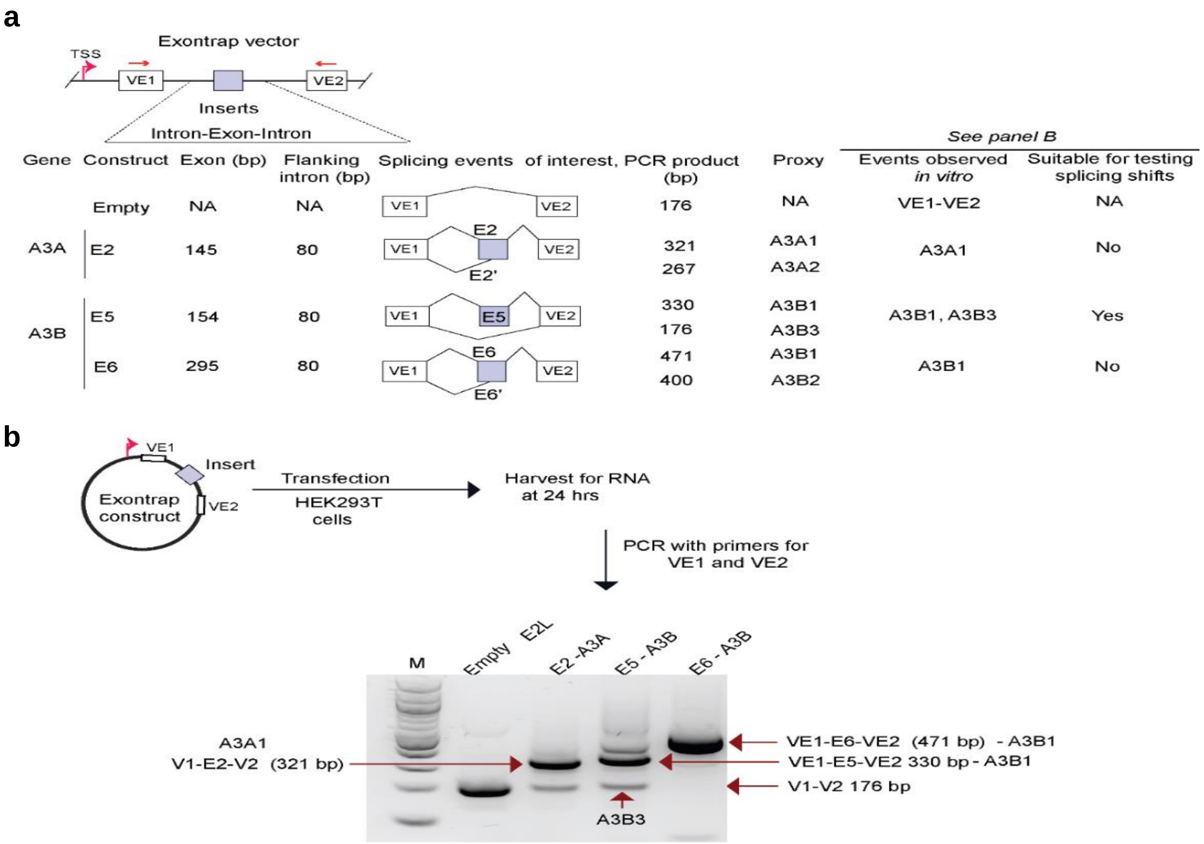

Supplementary Fig. 6. Analysis of alternative splicing in A3A and A3B with Exontrap mini-gene assays.

a) Schematic representation of the Exontrap strategy - the alternatively spliced exons (E) of A3A (exon 2) and A3B (exons 5 and 6) were cloned into an Exontrap vector, which carries a transcription start site (TSS) and vector exons VE1 and VE2. DNA fragments are cloned into insert position and tested for exon inclusion (trapping) between vector exons VE1 and VE2. Due to the large size of flanking introns (>1Kb), constructs were generated for each alternative exon with only 80 bp flanking intronic regions, assuming these sequences include essential cis-elements such as splice and branch point sites. The goal of the initial screening was to identify Exontrap constructs that generate predicted splicing events representing all A3A and A3B isoforms. A proxy for A3A1 is represented by splicing of vector exon VE1 with the insert exon E2 and then with vector exon VE2, as VE1- E2-VE2, while a proxy for A3A1 is represented by splicing of VE1 with E2 through a cryptic internal splicing site (E2'), as VE1- E2'-VE2. Proxies for A3B1 are represented by the inclusion of E5 or E6, as VE1- E5-VE2 or VE1- E6-VE2; a proxy for A3B2 is represented by splicing of VE1 with E6 through a cryptic splice site (E6'), as VE1- E6'-VE2, and a proxy for A3B3 in this context is represented by the exclusion of E5, as VE1-VE2.

b) Screening of Exontrap constructs transiently transfected into HEK293T cells for 24 hours. RNA was extracted, and PCR products were generated from cDNA with primers corresponding to vector exons VE1 and VE2 and resolved by agarose gel electrophoresis. Each band was cut out from the gel, purified and Sanger-sequenced to confirm the identity of splicing events. In HEK293T cells, only the E5 construct showed both splicing events of interest (representing

A3B1 and A3B3 transcripts) and was selected for further detailed analysis. M – 100 bp size marker.

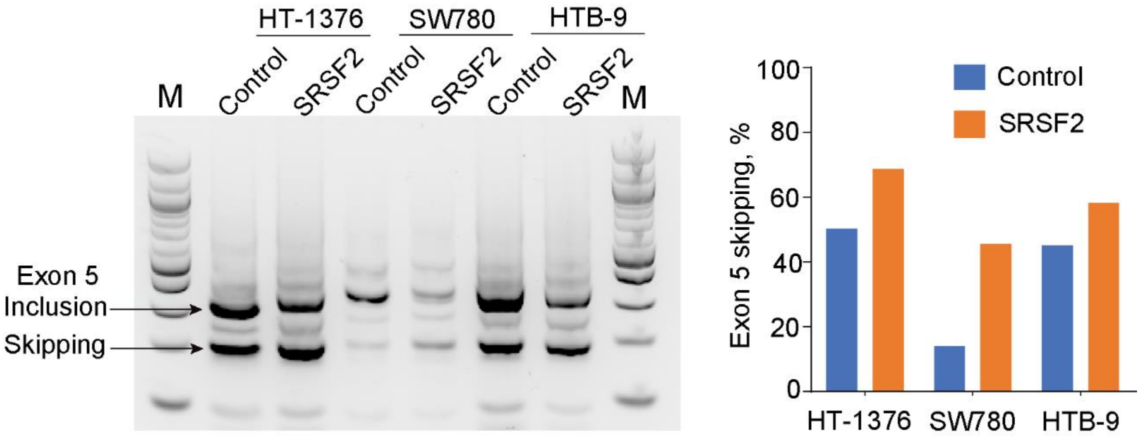

Supplementary Fig. 7. Overexpression of SRSF2 increases A3B exon 5 skipping in cancer cell lines.

Exontrap mini-gene E5 with exon 5 of A3B was transiently transfected into bladder cancer cell lines – HT-1376, SW780, and HTB-9 together with the SRSF2 expression construct or alone (control). Cells were harvested after 24 hrs and splicing products corresponding to exon 5 inclusion (A3B1-type transcript) vs. skipping (A3B3-type transcript) were analyzed by RT-PCR and agarose gel electrophoresis. Band intensity was quantified using Image Lab software (BioRad) and plotted as % of exon 5 skipping of the combined expression of A3B1 and A3B3 transcripts in each sample. Shown are representative results of one of the three independent experiments.

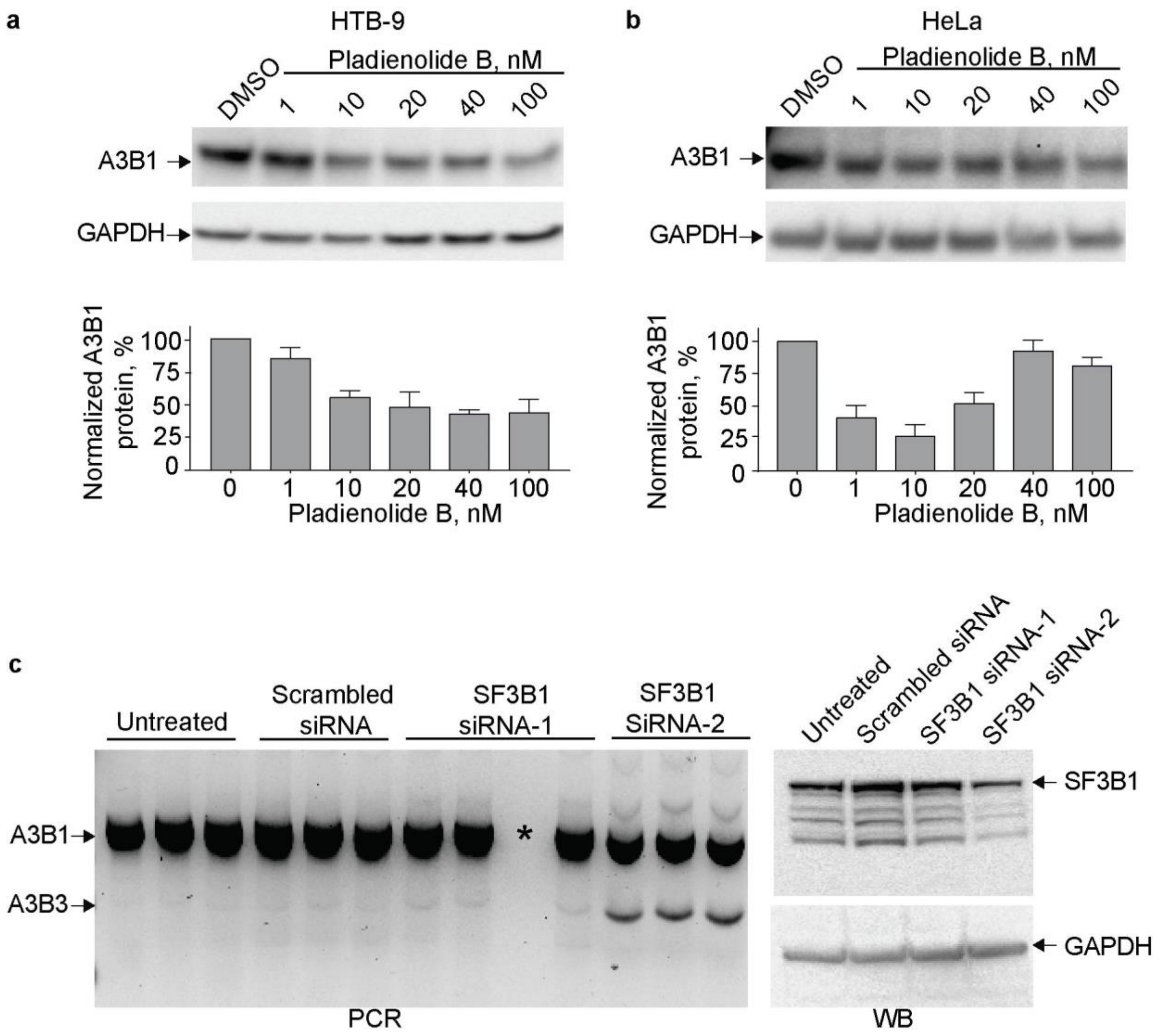

Supplementary Fig. 8. Effects of pladienolide B treatment and siRNA-SF3B1 knockdown on A3B exon 5 skipping in cancer cell lines.

a, b) A3B1 protein levels in HTB-9 **(a)** and HeLa cells **(b)** treated with pladienolide B for 36 hrs. Western blot (WB) analysis shows 20-70% reduction of A3B1 protein levels in cells treated with different concentrations of pladienolide B. Protein levels were determined by densitometry (ImageJ) and graphed as a percentage compared to A3B1 expression levels in the cells treated with DMSO (vehicle), after normalization by GAPDH expression (loading control). All bar graphs show means ± SD of biological triplicates. The effect is observed only in HTB-9 but not in HeLa cells. **c)** siRNA-SF3B1 knockdown in HT-1376 cells. Left panel: RT-PCR products analyzed by agarose gel electrophoresis showing the change in A3B exon 5 skipping in cells transfected for 36 hrs with SF3B1 siRNA-1 or 2, compared to untransfected cells and cells treated with scrambled siRNA (negative control), all in biological triplicates. Right panel: WB analysis with an anti-SF3B1 antibody confirms the S3FB1 protein knockdown, which was more successful using siRNA-2, judging from the RT-PCR results for A3B1 exon 5 skipping and WB analysis for SF3B1 expression.* - empty lane.

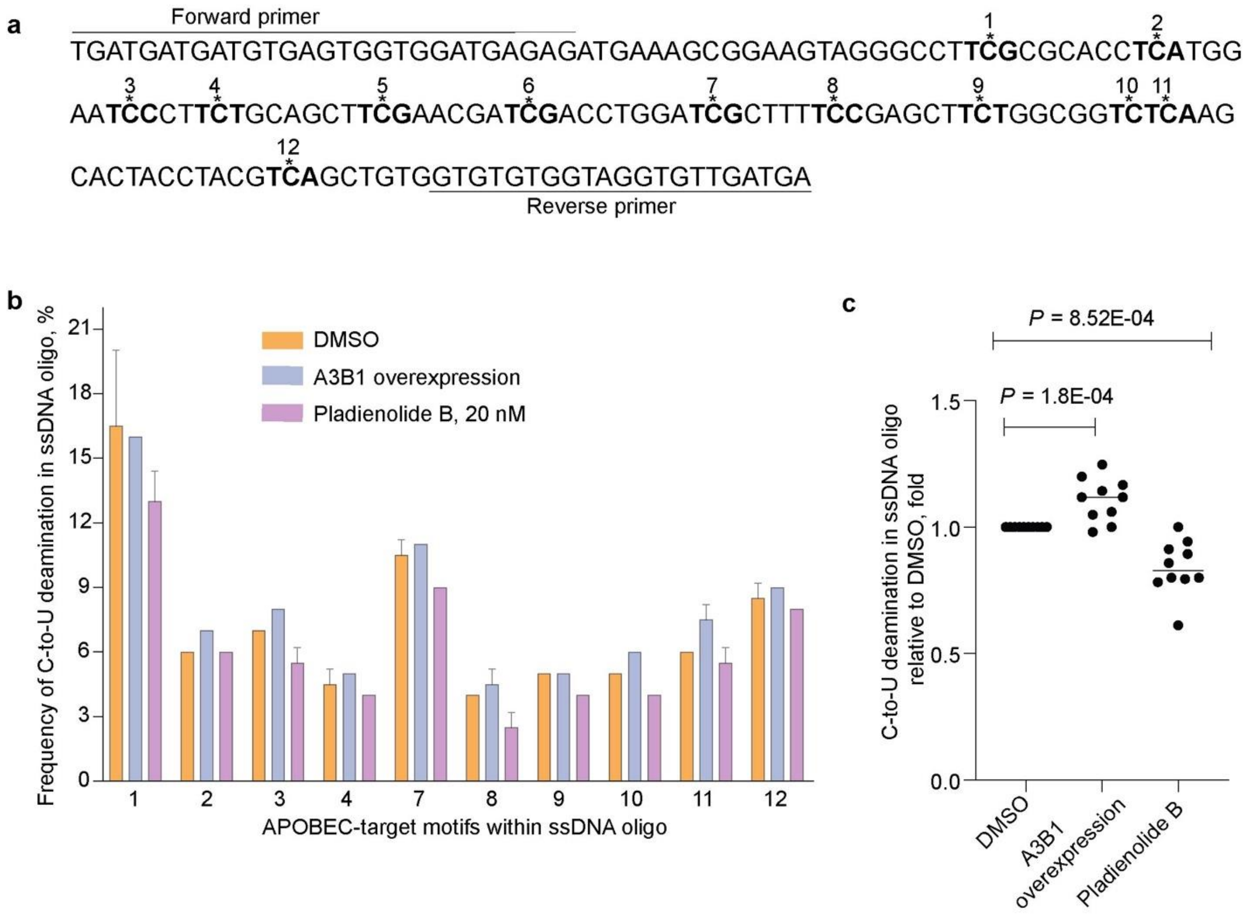

Supplementary Fig. 9. Cell-based cytosine deamination assay.

a) The sequence of the 169-bp ssDNA oligo with 12 APOBEC-target motifs (TC̲N) shown in bold and target cytosines indicated by asterisks and motif numbers (1-12). **b)** Frequencies (%) of cytosine deamination (C->U) events within each APOBEC motif in different experimental conditions. Bladder cancer HT-1376 cells were nucleofected with the ssDNA oligo alone or together with A3B1-expressing plasmid (positive control); after 8 hours, cells transfected only with ssDNA oligo were treated with DMSO or pladienolide B (20 nM) for 64 hours. The ssDNA oligos were recovered from all the cells, PCR-amplified and deep-sequenced (see Materials and Methods). All bar graphs show means ± SD of biological duplicates. APOBEC motifs #5 and #6 were excluded from the analysis due to low coverage of this region by the short (76 bp) paired sequencing reads not reaching the middle of the 169-bp PCR product. **c**) The ratios of cytosine deamination events within all APOBEC motifs in the ssDNA oligo in indicated experimental conditions compared to DMSO-treated cells. Each dot represents the ratio of C->U deamination at one of the ten APOBEC-target motifs and center lines represents mean values. Overall, these ratios were significantly higher in A3B1-overexpressing cells and lower in pladienolide B - treated cells compared to DMSO-treated control cells. *P*-values are for the Student's t-test.

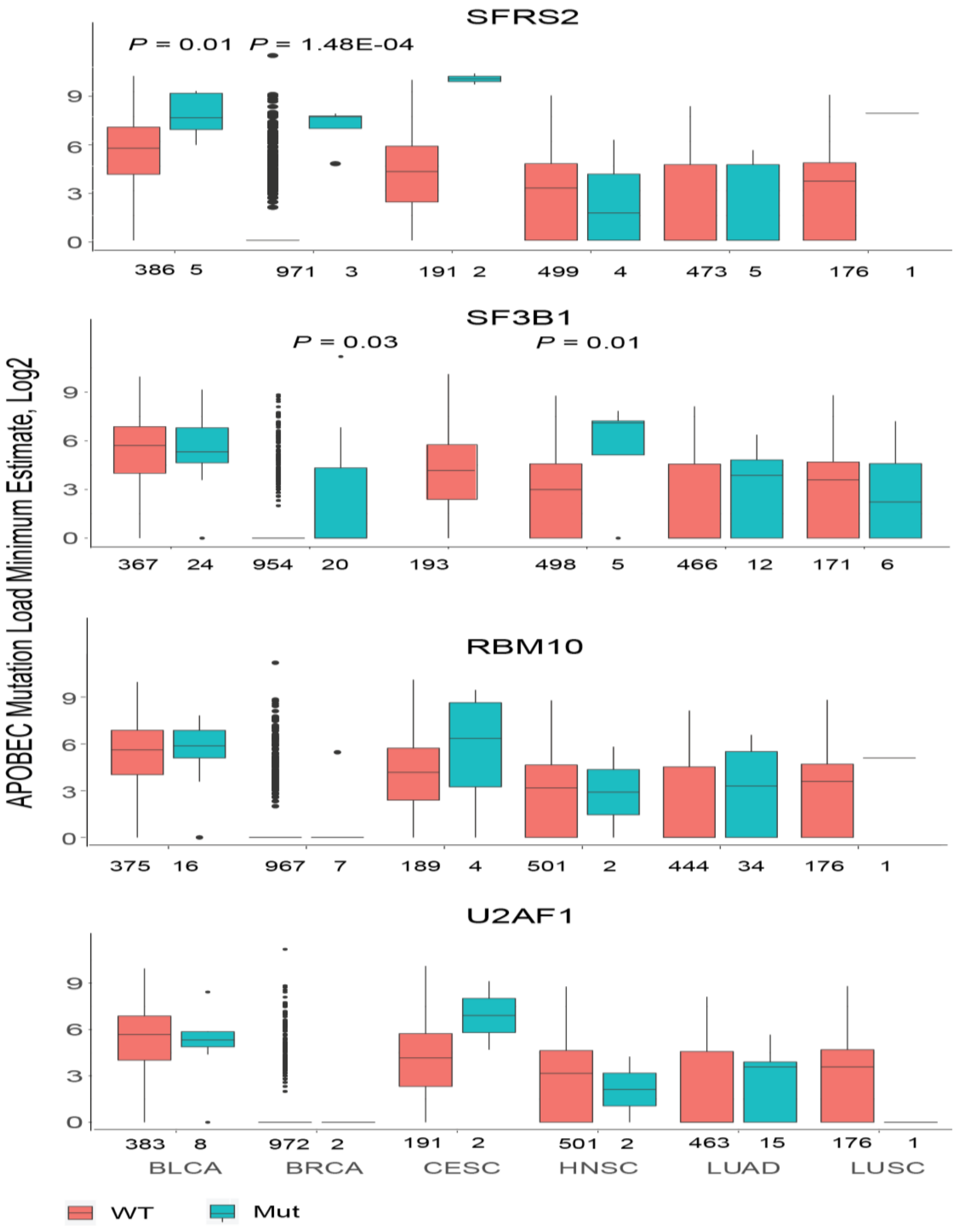

Supplementary Fig. 10. Analysis of APOBEC-mediated mutagenesis in six TCGA cancer types in relation to mutations in select splicing factors.

The analysis included nonsense, missense, splice site, and in-frame deletion/insertion mutations in splicing factors SFRS2, SF3B1, RBM10, and U2AF1, considering only driver mutations[7]. A trend for a higher load of APOBEC-signature mutations is observed in some cancer types with mutations in SFRS2 and SF3B1. Box-and-whisker plots represent median (center line), box (25th–75th interquartile range), whiskers (5th–90th percentile range) and outliers as individual data points. *P* values are for non-parametric, two-sided Mann–Whitney U tests.

Supplementary Fig. 11. Unprocessed blots and gels (pages 19 - 26)

Fig. 2e

Deamination assay gel

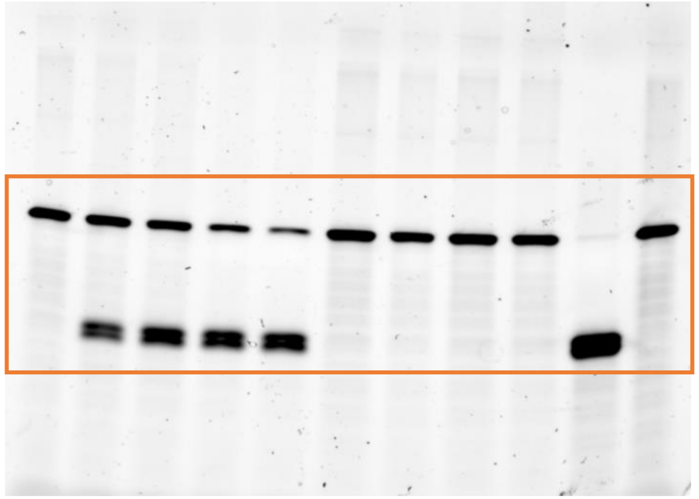

Western Blot: Anti-DDK tag

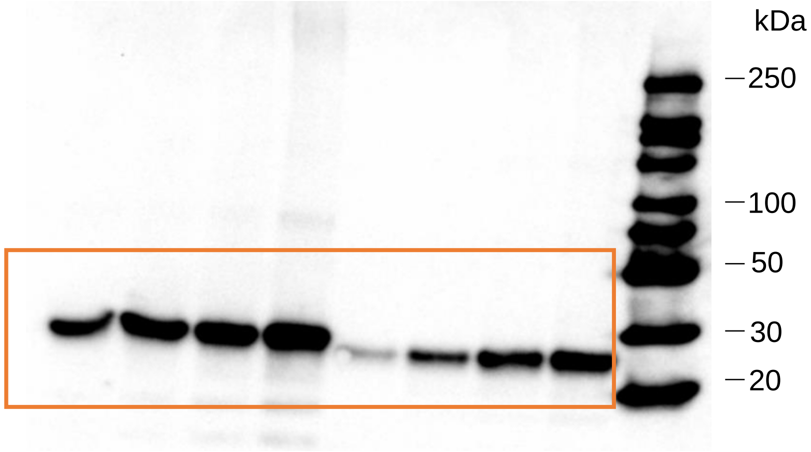

Fig. 2f

Deamination assay gel

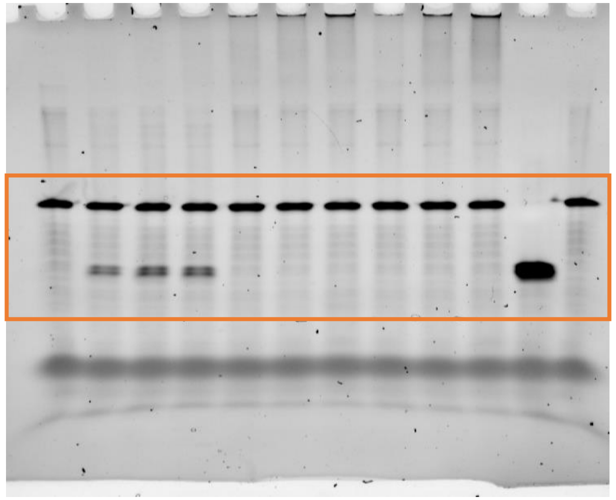

Western Blot: Anti-DDK tag

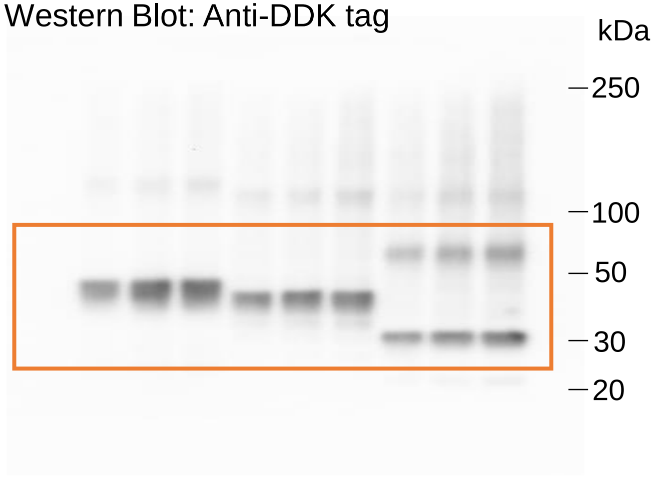

Fig. 2g

Western Blot: Anti-HA tag

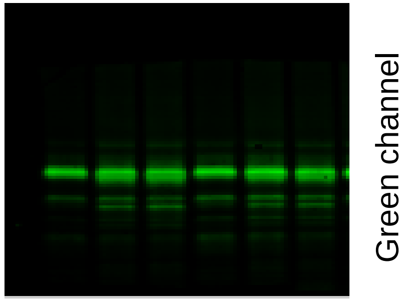

Western Blot: Anti-Tubulin

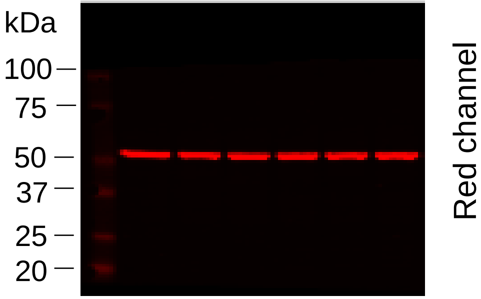

Fig. 4a

Agarose gels

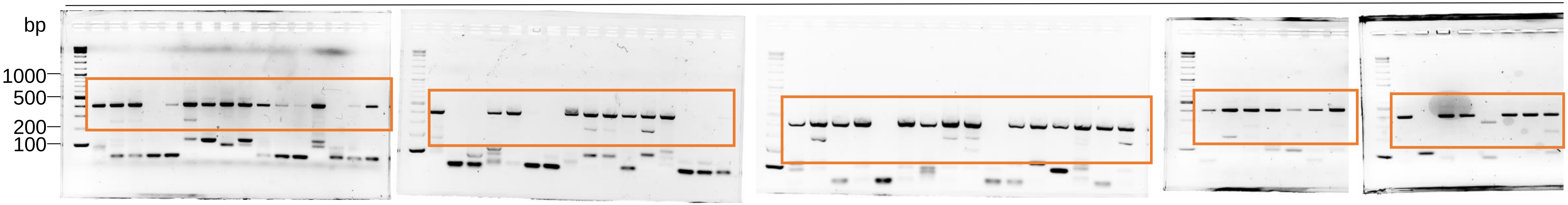

Fig. 4b

Western Blots: Anti-A3B

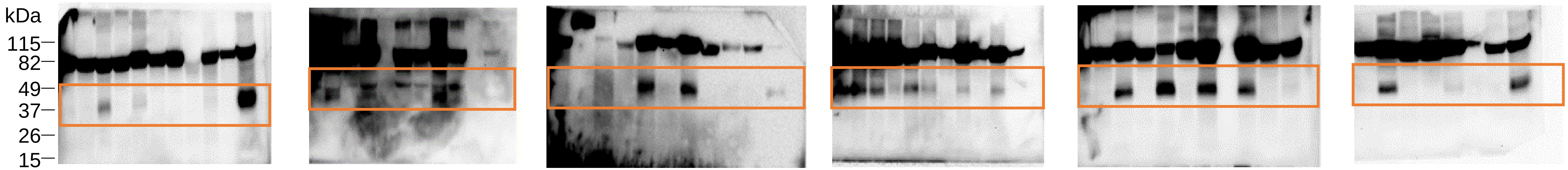

Western Blots: Anti-GAPDH

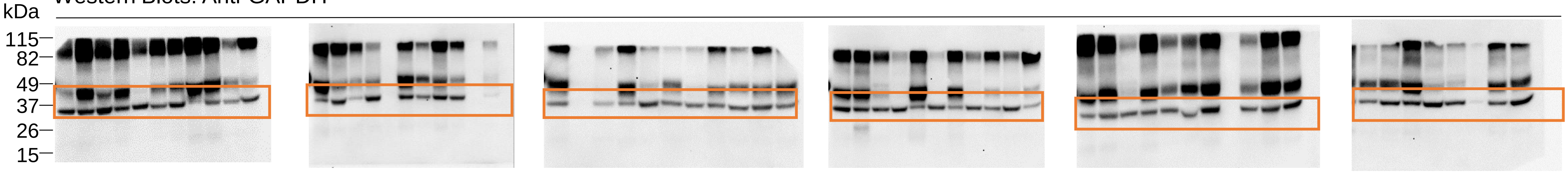

Fig. 5b

Agarose gel for mini-gene exon 5 splicing

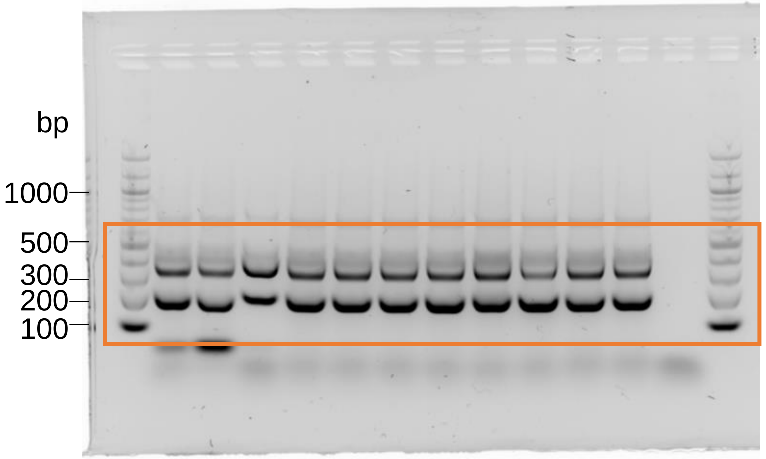

Agarose gel for control (ctrl)

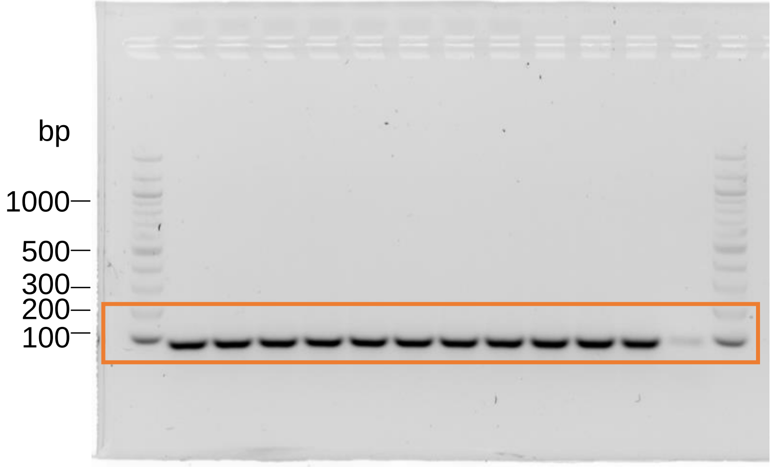

Fig. 5c

Agarose gel for mini-gene exon 5 splicing in cell lines

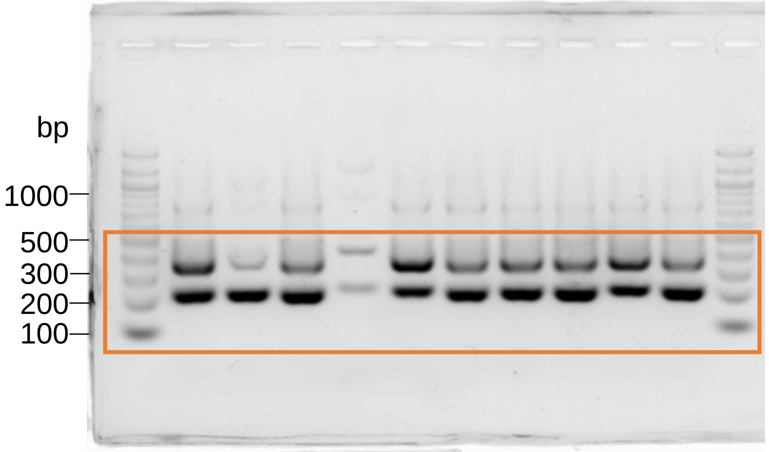

Agarose gel for control (ctrl)

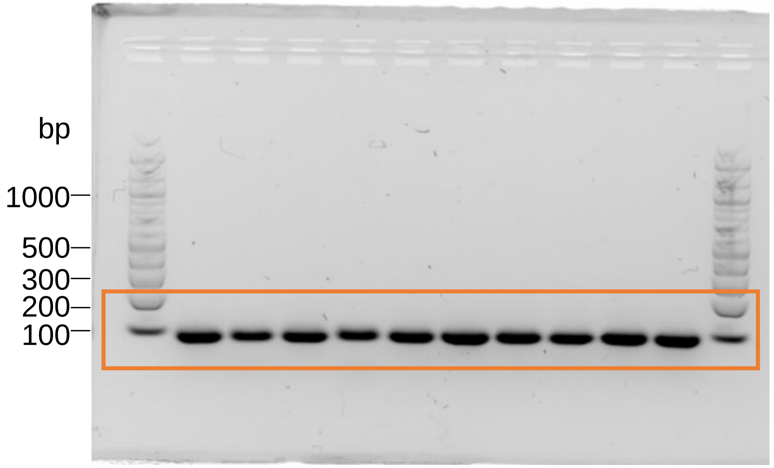

Fig. 5f

Agarose gel

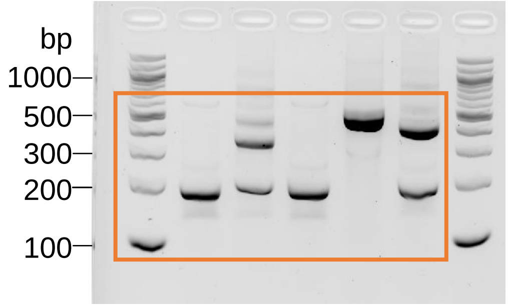

Fig. 6e

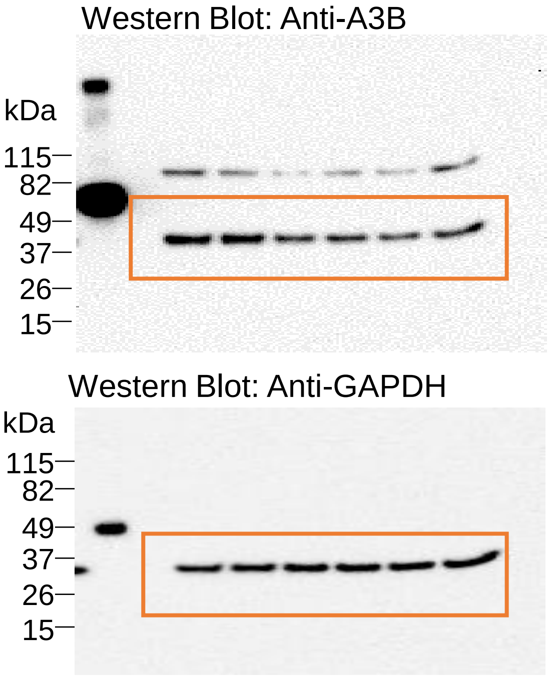

Fig. 6g

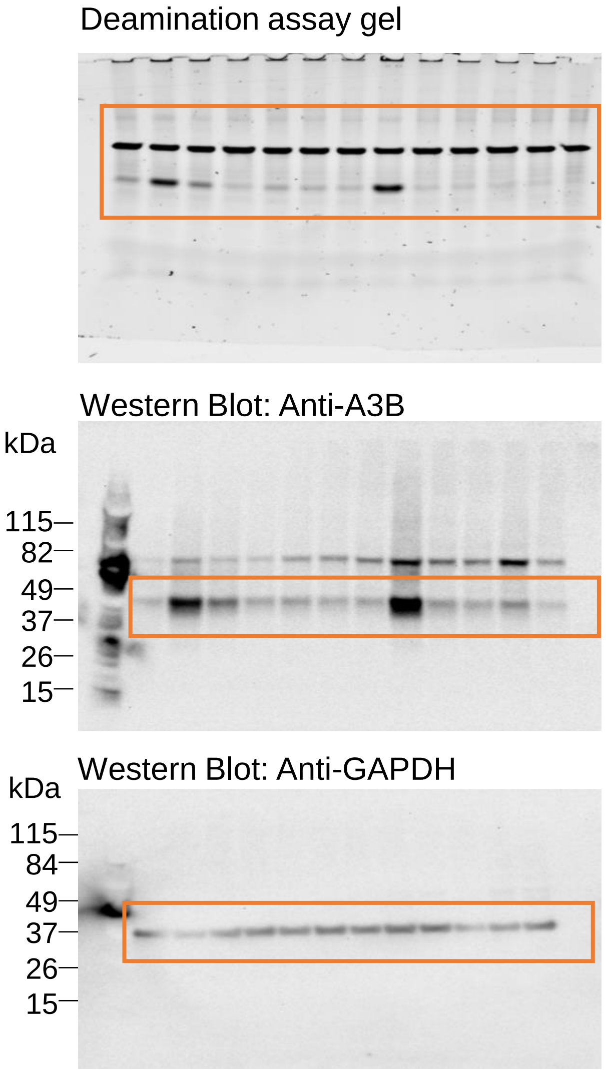

Fig. 6h

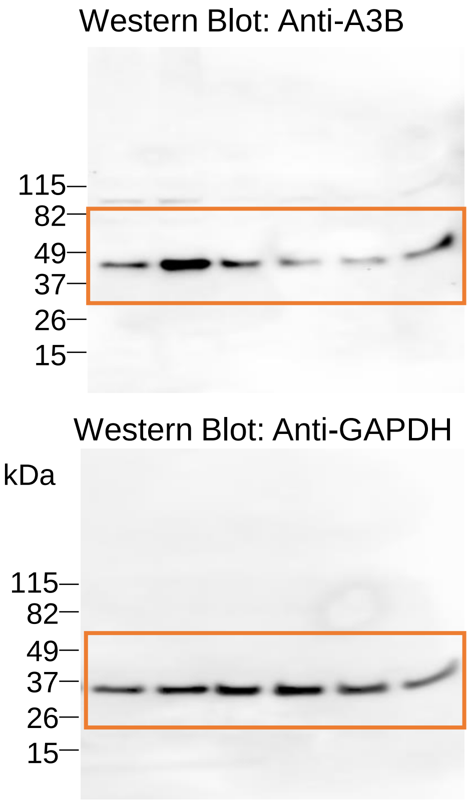

Fig. 7b

Agarose gel

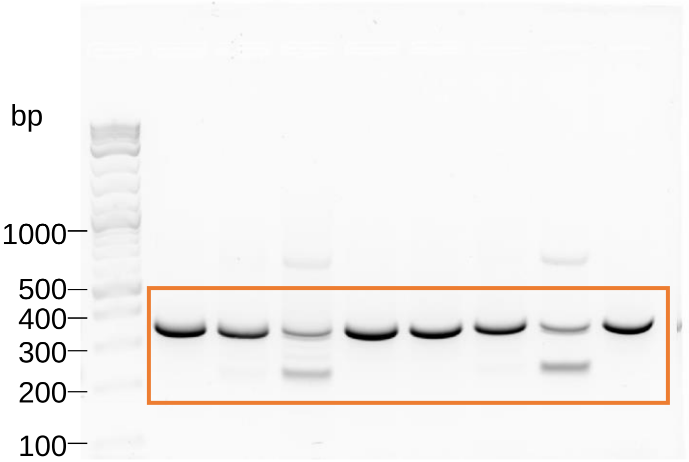

Supplementary Fig. 2a

Deamination assay gel

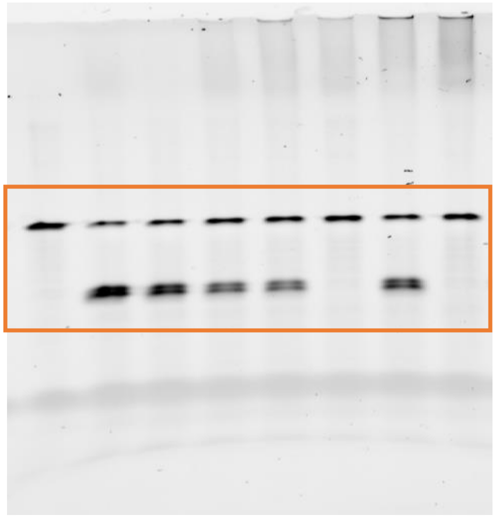

Western Blot: Anti-DDK tag

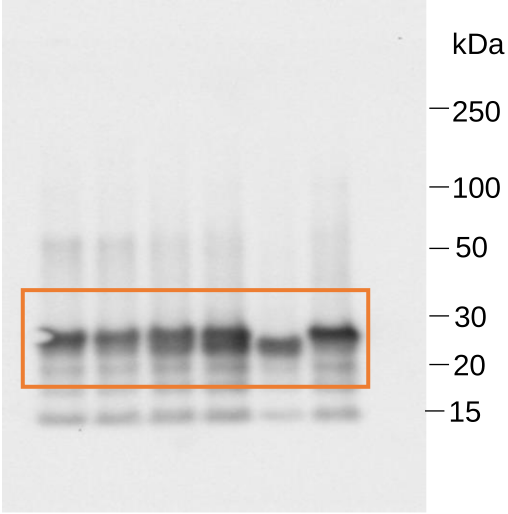

Supplementary Fig. 2b

Deamination assay gel

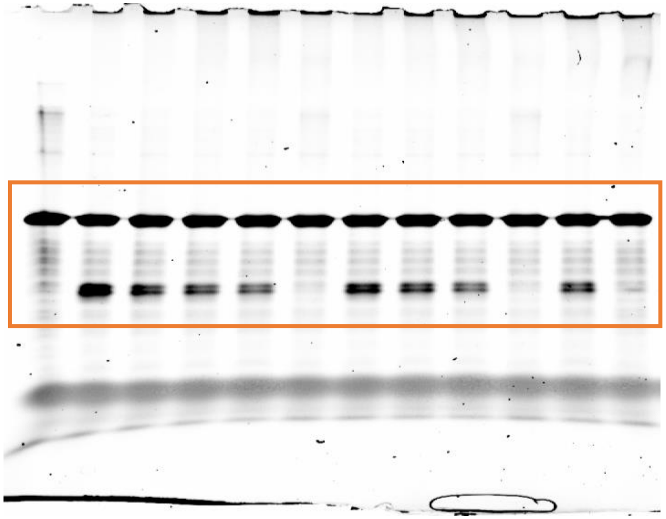

Western Blot: Anti-DDK tag

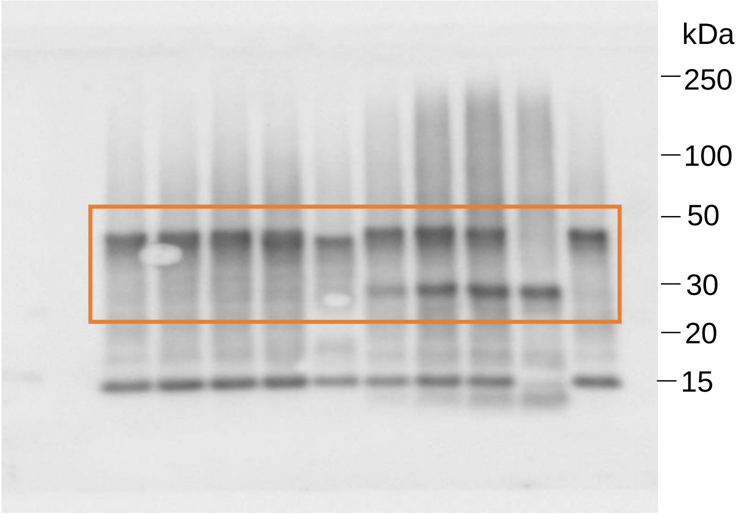

Supplementary Fig. 3b

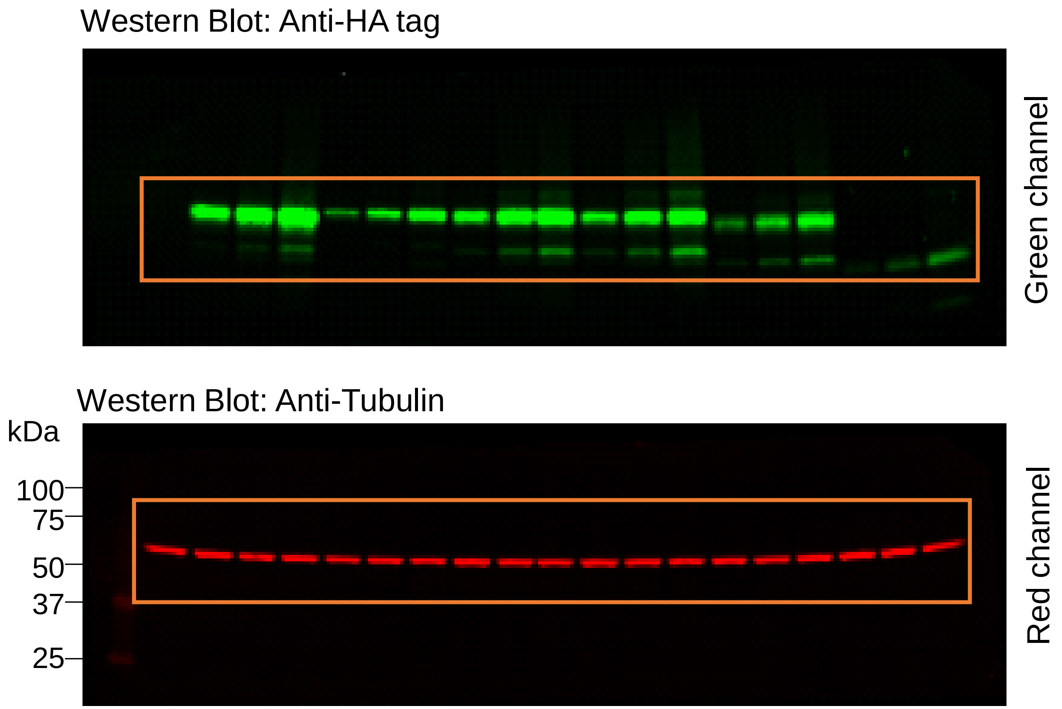

Supplementary Fig. 3c

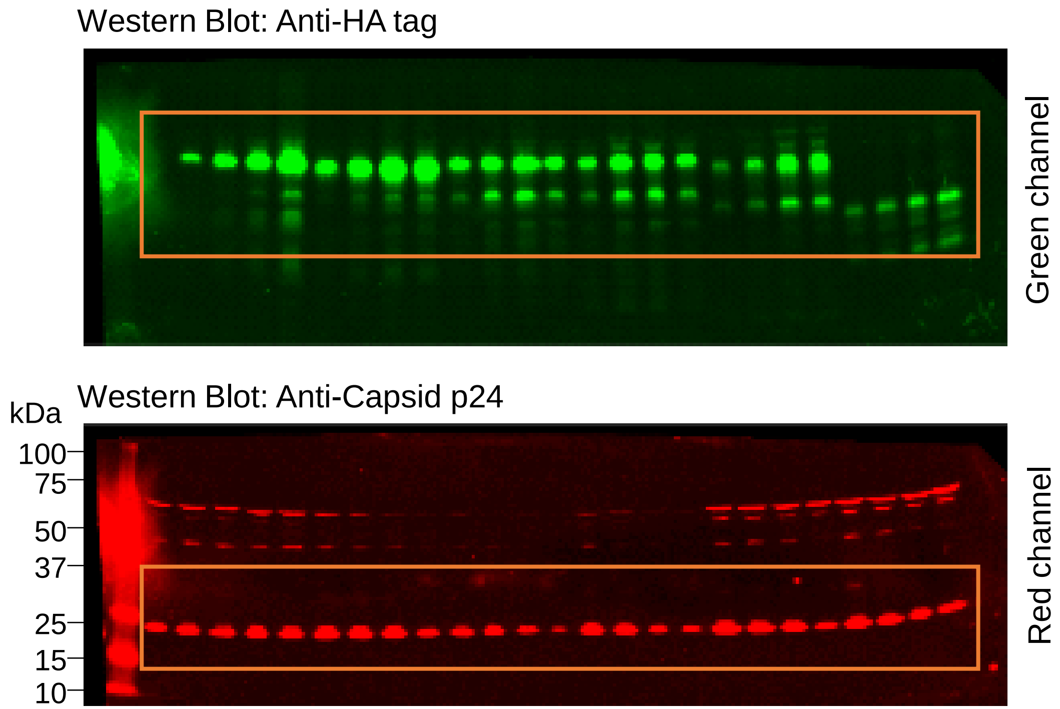

Supplementary Fig. 8a

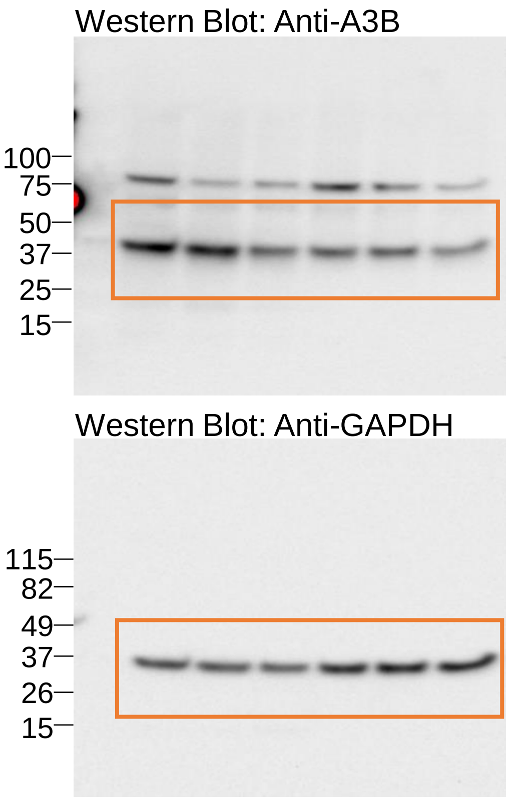

Supplementary Fig. 8b

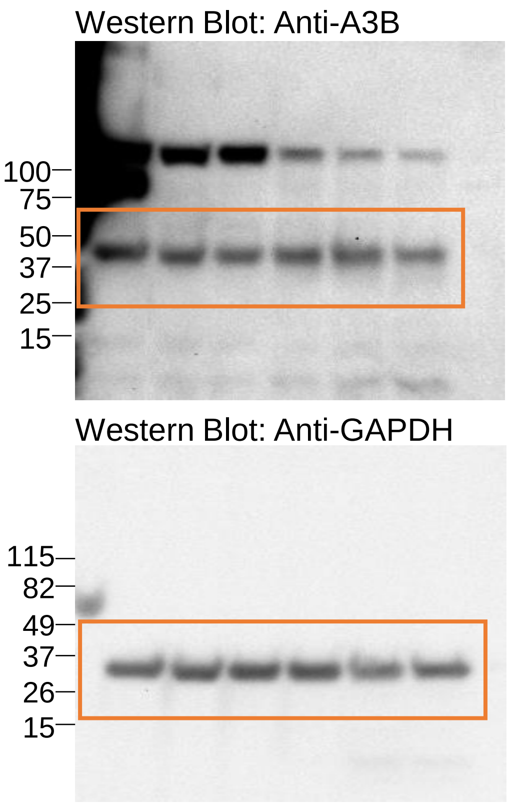

Supplementary Fig. 8c

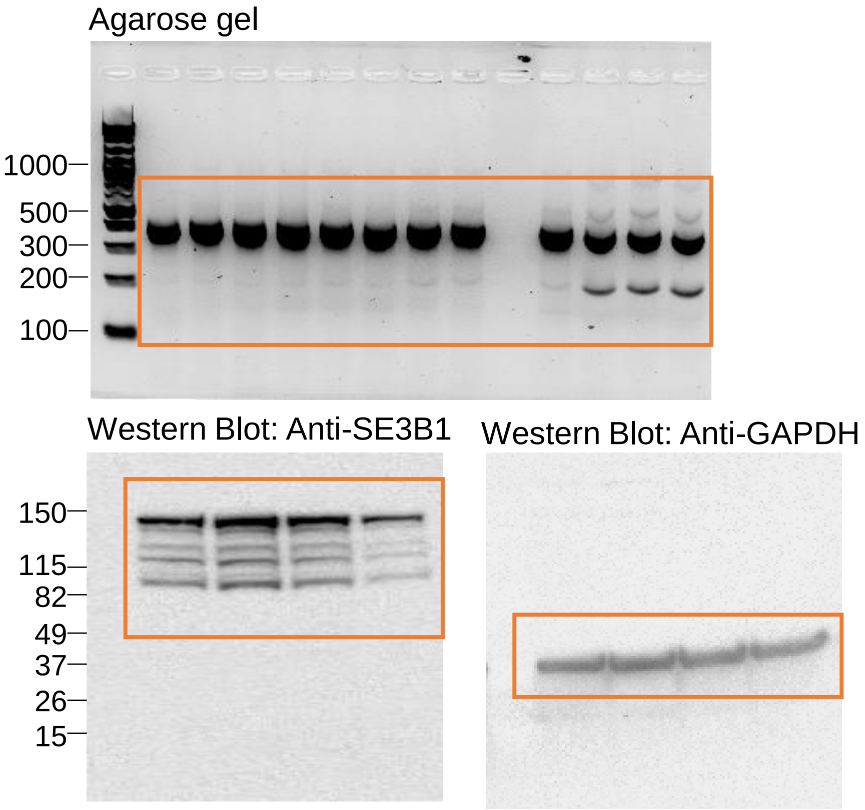

NOTES

Supplementary Note 1. Analysis of A3A and A3B transcripts based on exon-exon junctions and total expression using qRT-PCR and RNA-seq

```
A3A      ------------------------------------------------------------      0
A3B      ATGAATCCACAGATCAGAAATCCGATGGAGCGGATGTATCGAGACACATTCTACGACAAC      60

A3A      ------------------------------------------------------------      0
A3B      TTTGAAAACGAACCCATCCTCTATGGTCGGAGCTACACTTGGCTGTGCTATGAAGTGAAA      120

A3A      ------------------------------------------------------------      0
A3B      ATAAAGAGGGGCCGCTCAAATCTCCTTTGGGACACAGGGGTCTTTCGAGGCCAGGTGTAT      180

A3A      ------------------------------------------------------------      0
A3B      TTCAAGCCTCAGTACCACGCAGAAATGTGCTTCCTCTCTTGGTTCTGTGGCAACCAGCTG      240

A3A      ------------------------------------------------------------      0
A3B      CCTGCTTACAAGTGTTTCCAGATCACCTGGTTTGTATCCTGGACCCCCTGCCCGGACTGT      300

A3A      ------------------------------------------------------------      0
A3B      GTGGCGAAGCTGGCCGAATTCCTGTCTGAGCACCCCAATGTCACCCTGACCATCTCTGCC      360

A3A      ------------------------------------------------------------      0
A3B      GCCCGCCTCTACTACTACTGGGAAAGAGATTACCGAAGGGCGCTCTGCAGGCTGAGTCAG      420

A3A      ------------------------------------------------------------      0
A3B      GCAGGAGCCCGCGTGACGATCATGGACTATGAAGAATTTGCATACTGCTGGGAAAACTTT      480

A3A      ------------------------------------------------------------      0
A3B      GTGTACAATGAAGGTCAGCAATTCATGCCTTGGTACAAATTCGATGAAAATTATGCATTC      540

                                 78.8.3% identity between A3A-Ex2 and A3B-Ex5
A3A      ATGGAAGCCAGCCCAGCATCCGGGCCCAGACACTTGATGGATCCACACATATTCACTTCC      60
A3B      CTGCACCGCACGCTAAAGGAGATTCTCAGATACCTGATGGATCCAGACACATTCACTTTC      600
          ** *   **  * *         * **** ** *********** *** ******** *

A3A      AACTTTAACAATG---------GCATTGGAAGGCATAAGACCTACCTGTGCTACGAAGTG      111
A3B      AACTTTAATAATGACCCTTTGGTCCTTCGACGGCGCCAGACCTACTTGTGCTATGAGGTG      660
         ******** ****          * ** ** ***   ******** ******* ** ***

A3A      GAGCGCCTGGACAATGGCACCTCGGTCAAGATGGACCAGCACAGGGGCTTTCTACACAAC      171
A3B      GAGCGCCTGGACAATGGCACCTGGGTCCTGATGGACCAGCACATGGGCTTTCTATGCAAC      720
         ********************** ****  ************** **********  ****

          99.3% identity between A3A-Ex3 and A3B-Ex6
A3A      CAGGCTAAGAATCTTCTCTGTGGCTTTTACGGCCGCCATGCGGAGCTGCGCTTCTTGGAC      231
A3B      GAGGCTAAGAATCTTCTCTGTGGCTTTTACGGCCGCCATGCGGAGCTGCGCTTCTTGGAC      780
          ***********************************************************

A3A      CTGGTTCCTTCTTTGCAGTTGGACCCGGCCCAGATCTACAGGGTCACTTGGTTCATCTCC      291
A3B      CTGGTTCCTTCTTTGCAGTTGGACCCGGCCCAGATCTACAGGGTCACTTGGTTCATCTCC      840
         ************************************************************

A3A      TGGAGCCCCTGCTTCTCCTGGGGCTGTGCCGGGGAAGTGCGTGCGTTCCTTCAGGAGAAC      351
```

```
A3B     TGGAGCCCCTGCTTCTCCTGGGGCTGTGCCGGGGAAGTGCGTGCGTTCCTTCAGGAGAAC     900
        ************************************************************

A3A     ACACACGTGAGACTGCGTATCTTCGCTGCCCGCATCTATGATTACGACCCCCTATATAAG     411
A3B     ACACACGTGAGACTGCGCATCTTCGCTGCCCGCATCTATGATTACGACCCCCTATATAAG     960
        ***************** ******************************************

A3A     GAGGCACTGCAAATGCTGCGGGATGCTGGGGCCCAAGTCTCCATCATGACCTACGATGAA     471
A3B     GAGGCGCTGCAAATGCTGCGGGATGCTGGGGCCCAAGTCTCCATCATGACCTACGATGAG     1020
        ***** *****************************************************
```

94.8% identity between A3A-Ex4 and A3B-Ex7

```
A3A     TTTAAGCACTGCTGGGACACCTTTGTGGACCACCAGGGATGTCCCTTCCAGCCCTGGGAT     531
A3B     TTTGAGTACTGCTGGGACACCTTTGTGTACCGCCAGGGATGTCCCTTCCAGCCCTGGGAT     1080
        *** ** ******************** *** ****************************

A3A     GGACTAGATGAGCACAGCCAAGCCCTGAGTGGGAGGCTGCGGGCCATTCTCCAGAATCAG     591
A3B     GGACTAGAGGAGCACAGCCAAGCCCTGAGTGGGAGGCTGCGGGCCATTCTCCAGAATCAG     1140
        ******** ***************************************************

A3A     GGAAACTGA          600
A3B     GGAAACTGA          1149
        *********
```

I). High homology between coding sequences of *A3A* and *A3B* genes

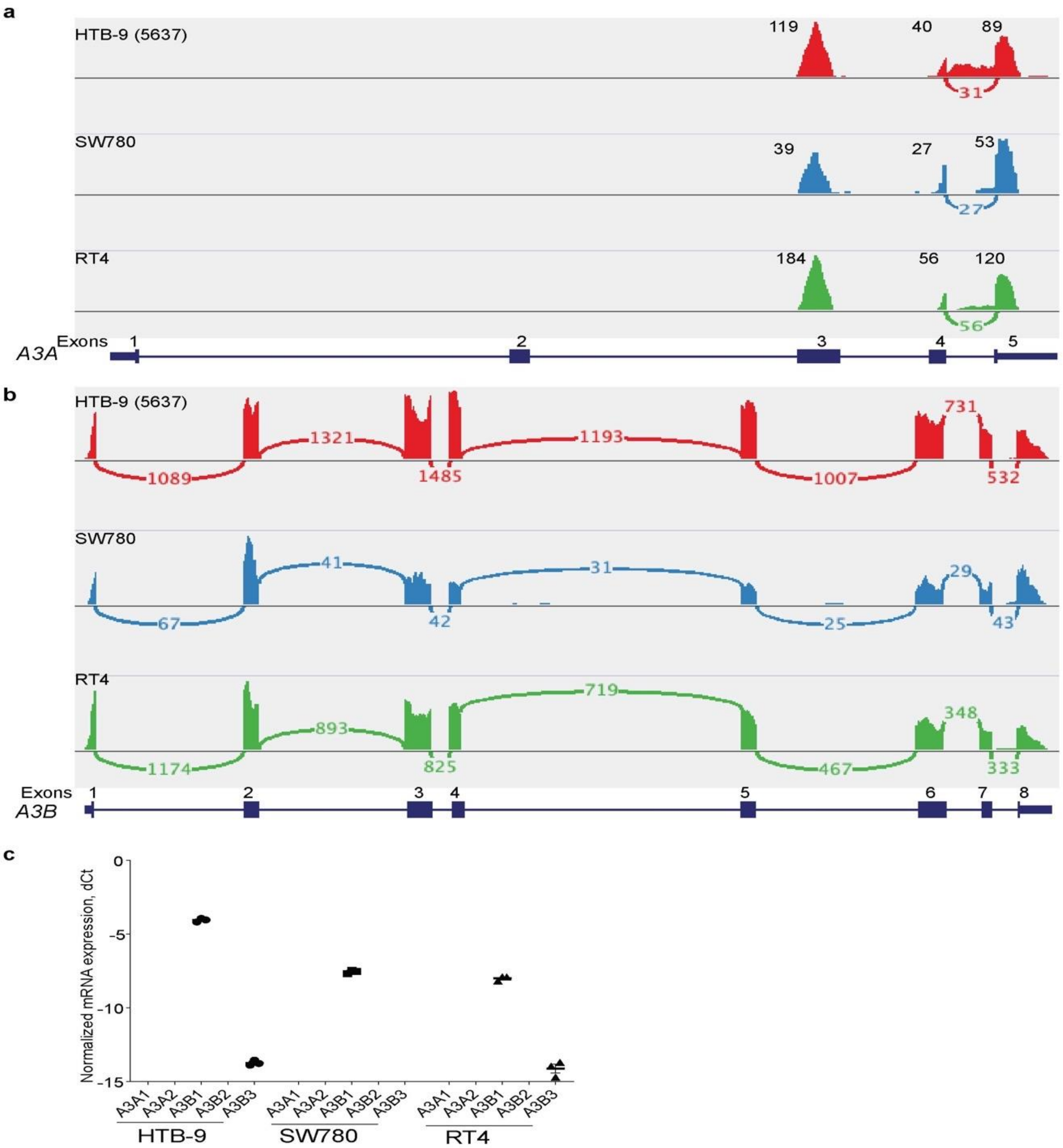

II). Due to high sequence homology, *A3B* RNA-seq reads misalign to *A3A*

Expression of *A3A* and *A3B* genes visualized as IGV Sashimi plots based on RNA-seq BAM files for bladder cancer cell lines HTB-9 (referred to as 5637 in CCLE), SW780, and RT4, all from Broad Institute Cancer Cell Line Encyclopedia (CCLE). **a**) Within *A3A*, RNA-seq reads align only to exons 3-5; **b**) Within *A3B*, RNA-seq reads align to all exons. **C**) qRT-PCR analysis in corresponding cell lines shows high expression of *the* A3B1 isoform and weak expression of the A3B3 isoform, while no expression of the *A3A* isoforms. When expression of *A3B* is higher than of *A3A*, *A3B RNA*-seq reads misalign to highly homologous exons 3-5 of *A3A* (see **part I**) and incorrectly represent *A3A* expression. Numbers on IGV plots indicate read counts.

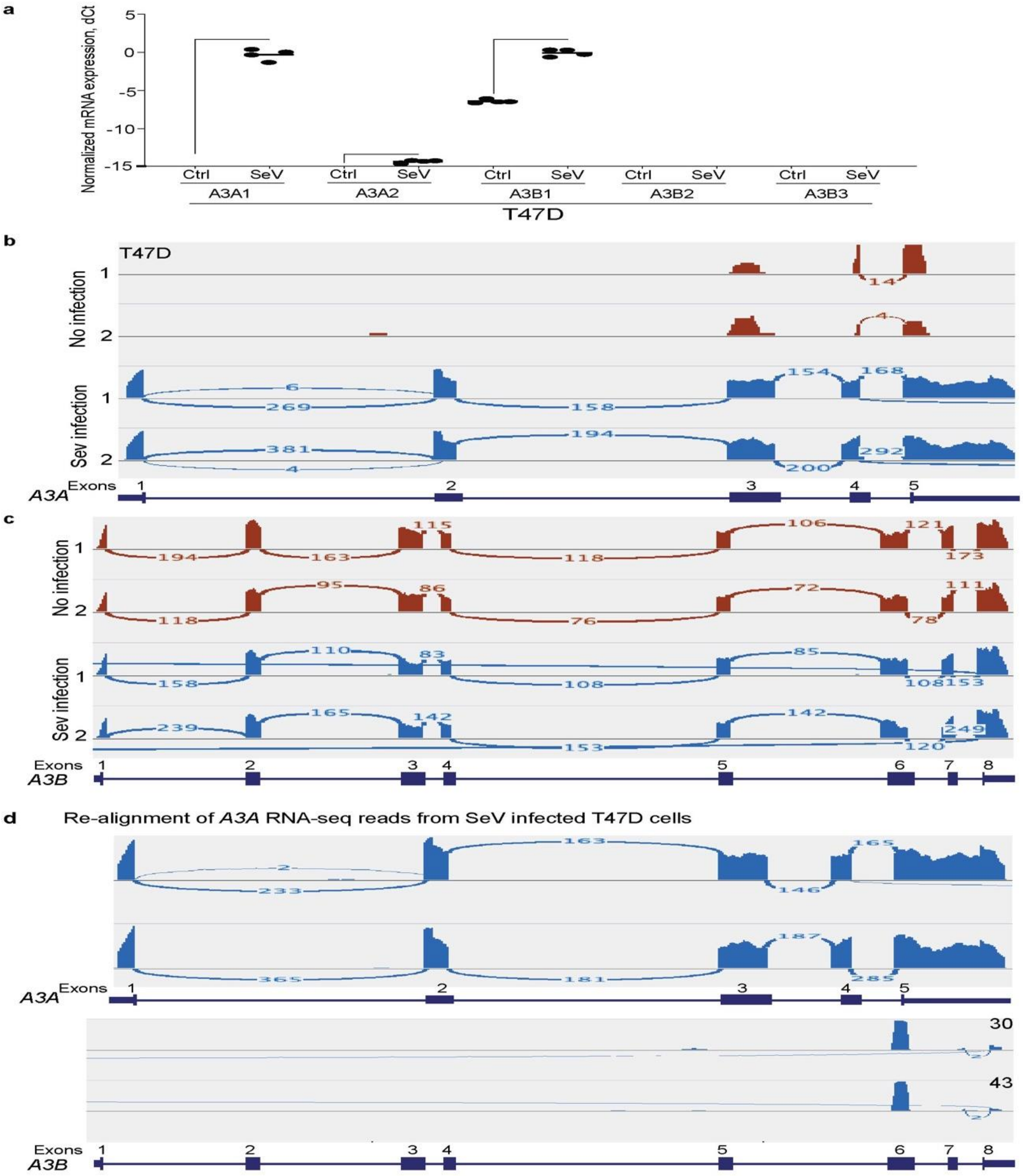

III). Due to high sequence homology, *A3A* RNA-seq reads misalign to *A3B*

a) qRT-PCR analysis of the *A3A* and *A3B* isoforms in a breast cancer cell line T47D in uninfected (control) conditions and in cells infected with Sendai virus (SeV), all in duplicates. After SeV infection, expression of *A3A1* was strongly induced, while A3B1 was moderately upregulated from baseline levels; **b** and **c**) IGV Sashimi plots showing RNA-seq expression profiles of *A3A* and *A3B* in corresponding samples, with *A3A* expression being contaminated by the misaligned A3B1 reads (see **part II** for details); **d**) Re-alignment of extracted A3A reads from SeV-infected T47D cells to the reference genome (hg38) shows misalignment of A3A1 RNA-seq reads to *A3B* (mostly to exon 6). When the expression of *A3A* is higher than of *A3B*, *A3A* RNA-seq reads misalign to highly homologous exon 6 of *A3B* (see **part I**) and incorrectly represent *A3B* expression.

Supplementary Note 2. Evaluation of the available antibodies for the detection of A3A and A3B protein isoforms

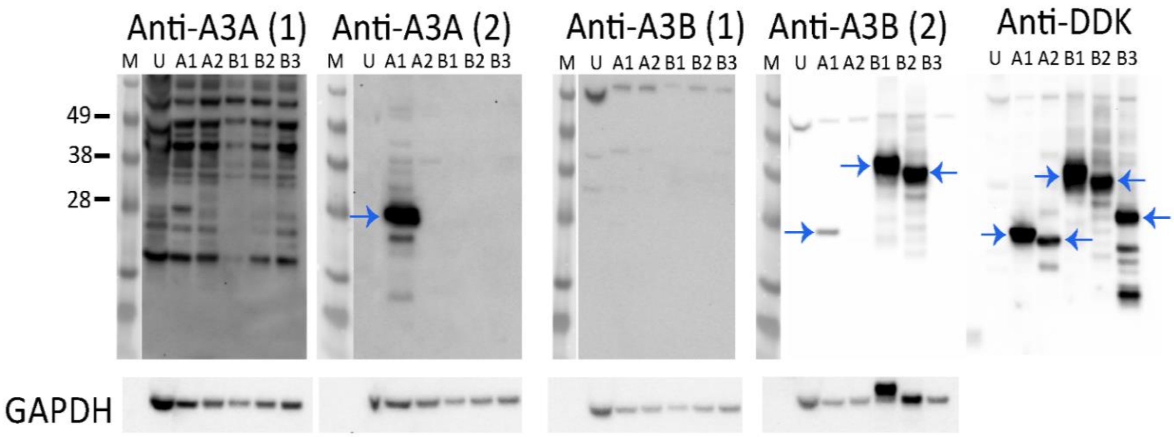

Expression constructs for recombinant Myc-DDK-tagged A3A and A3B protein isoforms (A3A1, A3A2, A3B1, A3B2, and A3B3) were overexpressed in HEK293T cells for 24 hrs. Whole-cell protein lysates of transfected and untransfected (U) cells were resolved on 4-12% SDS PAGE gels. Protein expression was analyzed by Western blots with antibodies described in the table; arrows mark bands considered to be specific.

Ab	ID	Company	Dilution	Comment
A3A (1)	orb19763	Biorbyt	1:500	Not specific
A3A (2)	HPA043237	Millipore Sigma	1:500	Detects only A3A1
A3B (1)	ab191695	Abcam	1:500	No detection
A3B (2)	ab184990	Abcam	1: 500	Detects A3B1 and A3B2 but not A3B3. Some weak detection of A3A2 as well
Anti-DDK	MAB3118	Millipore Sigma	1:1000	Detects all Myc-DDK-tagged proteins
Anti-SF3B1	ab172634	Abcam	1:1000	Detects SF3B1
GAPDH	ab9485	Abcam	1:1000	Loading control
mouse-IgGκ BP-HRP	sc-516102	Santa Cruz Biotechnology	1:5000	Secondary ab
Anti-α-tubulin	T9026	Sigma	1:10,000	Detects α-tubulin
Anti-HA	3724	Cell signaling	1:5000	Detects HA tag
Anti-HIV-1 p24	6458	Through NIH AIDS Reagent Program; from Dr. Michael Malim	1:10,000	Detects HIV-1 p24 protein

Anti-goat IgG-HRP	sc-2304	Santa Cruz Biotechnology	1:5000	Secondary ab
IRDye 800CW-labeled goat anti-rabbit	926-32211	LI-COR	1:10,000	Secondary ab
IRDye 680-labeled goat anti-mouse	926-68070	LI-COR	1:10,000	Secondary ab
Anti-rabbit IgG-HRP	7074	Cell Signaling	1:5000	Secondary ab

GAPDH blot with anti-A3B (2) antibody shows incomplete stripping of the original anti-A3B blot, which was not possible to exclude due to the close size of these bands.

Supplementary Note 3. Purification/enrichment of recombinant A3A and A3B protein isoforms

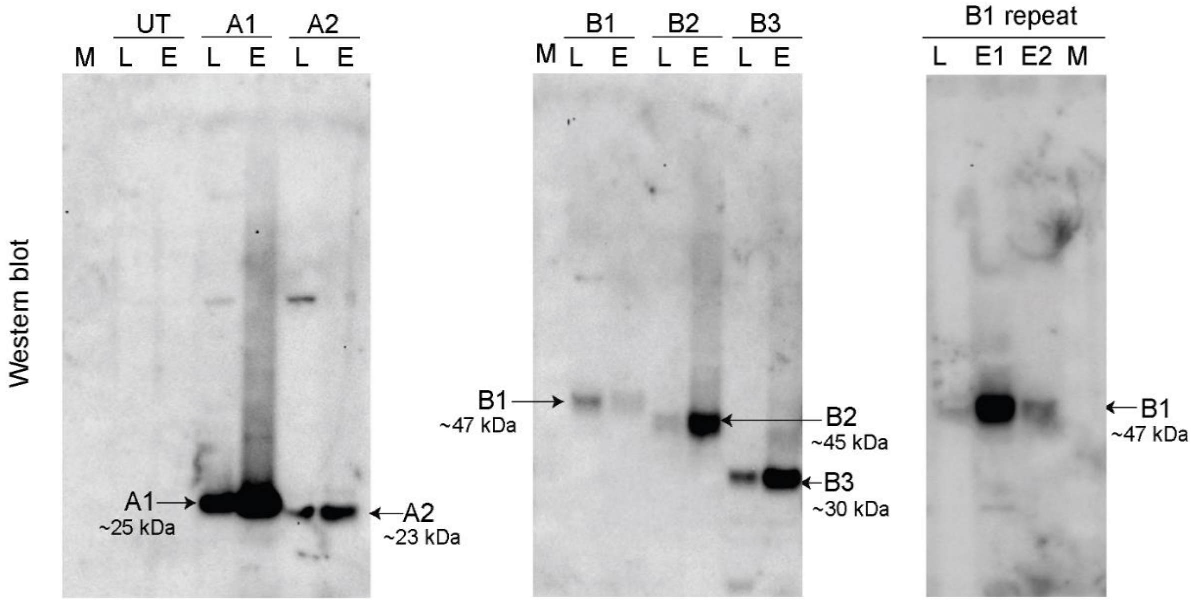

Recombinant Myc-DDK tagged A3A and A3B protein isoforms were purified as described in Materials and Methods. Semi-purified proteins eluted with Myc peptide were resolved on 4-12 % SDS PAGE gels and analyzed by Western blots with an anti-DDK antibody. Each eluate (E) from cells transfected with plasmids for Myc-DDK A3A and A3B isoforms or untransfected (UT) cells was compared with the corresponding cell lysates (L) to determine enrichment of the specific proteins.

Supplementary Note 4. Isoform-specific TaqMan expression assays for A3B transcripts

A. Sequences for primers and probes for custom TaqMan expression assays

Assay	Forward Primer	Reverse Primer	Probe, MGB (FAM-dye-labeled)
**A3B1**	ACCAGCACATGGGCTTTCTATG	AAAGAAGGAACCAGGTCCAAGAAG	CGAGGCTAAGAATCT
**A3B2**	GCTTTCTATGCAACGAGTTGGA	AGGAGAAGCAGGGGCTCC	CAGATCTACAGGGTCACTT
**A3B3**	CGCTAAAGGAGATTCTCAGGCTA	CAGGTCCAAGAAGCGCAGC	CTCTGTGGCTTTTACGGC
**A3B1&2 for NMD**	TGCTGGGAAAACTTTGTGTACAAT	ATGTGTCTGGATCCATCAGGTATCT	ATTCATGCCTTGGTACAAA

B. Characteristics of custom TaqMan expression assays

Assay \ Plasmid		A3A1	A3A2	A3B1	A3B2	A3B3	Comments
**A3B1**	R^2	0.70	0.97	**0.99**	0.94	0.70	Most specific for A3B1
	Ct value	32.52	27.84	**16.49**	36.05	34.67	
**A3B2**	R^2	0.79	0.77	0.86	**0.99**	0.99	Most specific for A3B2
	Ct value	36.93	39.15	36.11	**18.47**	26.19	
**A3B3**	R^2	0.98	0.52	0.98	0.36	**0.99**	Most specific for A3B3
	Ct value	39.13	37.94	30.63	37.23	**15.89**	
**A3B1&2**	R^2	0.42	0.38	**0.99**	**0.99**	0.99	Most specific for A3B1 and A3B2
	Ct value	33.34	33.40	**18.19**	**18.73**	26.47	

Expression assays were designed using Primer Express 3.0.1 software (ThermoFisher). The assays were tested using eight 4-fold linear dilutions (from 0.06 to 1000 ng/uL per reaction) of isoform-specific plasmids; R^2 coefficients correspond to dilution experiments. Results (Ct values) are shown for 1 ng/uL concentration of all plasmids. Based on analysis with UCSC tools (BLAT and *in silico* PCR), assays are most specific to the designated isoforms. Assays for A3B1, A3B2, and A3B3 can also detect other isoforms, but with much lower efficiency.

Supplementary Note 5. Correlation analysis between A3B exon 5 skipping and mRNA expression of select splicing factors in cell lines

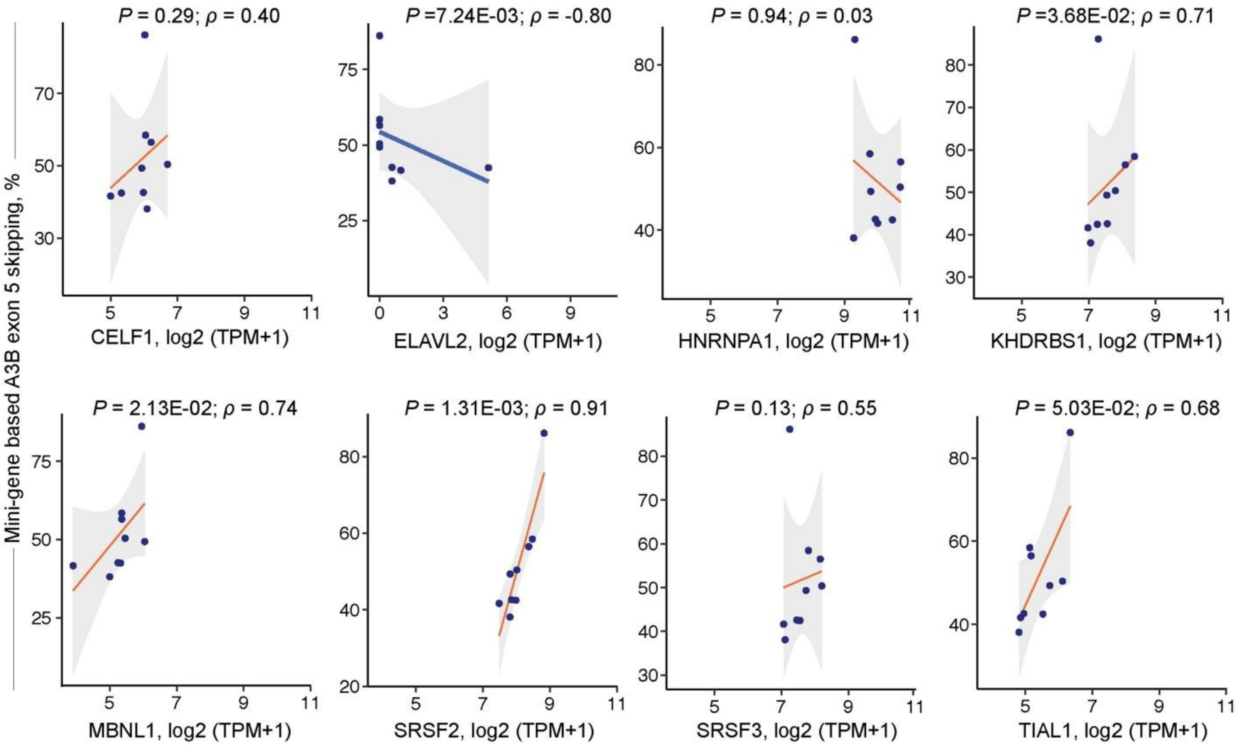

Correlation analysis between A3B exon 5 skipping was based on results of Exontrap mini-gene assays and mRNA expression of splicing factors in cell lines described in **Fig. 5c**. A3B exon 5 skipping ratios (as %) are based on the average values of three replicates presented in **Fig. 5c.** Expression of eight splicing factors was evaluated as log2 (TPM+1) values based on RNA-seq in nine cell lines - H460, HepG2, HT-1376, HTB-133, HTB-26, HTB-9, PA-TU-8988T, RT4 and SW-780. RNA-seq data for these cell lines was included in the Cancer Cell Line Encyclopedia (CCLE) and was acquired as BAM files from the GDC legacy archive (https://portal.gdc.cancer.gov/legacy-archive/search/f). HEK293T cell line was excluded from analysis as its RNA-seq data was not available in CCLE. Stringtie-2.1. 4. was used for whole-transcriptome level gene expression quantification using hg19 as the reference. Shown are Spearman correlation coefficients (ρ) with P values for two-sided tests. Shown linear trends, with shaded areas representing 95% confidence intervals. A3B exon 5 skipping was positively correlated with expression of KHDRBS1, MBNL1 and SRSF2 and negatively correlated with expression of ELAVL2. These results are in line with the overexpression model presented in **Fig. 5b** for SRSF2, which showed the strongest effect on skipping of A3B exon 5 in mini-gene assays. Overall, these data suggest that any of the splicing factors that bind within exon 5 may affect the expression levels of mutagenic A3B1 protein via controlling exon 5 splicing of its mRNA. These effects might be condition-dependent, contributed by variable levels of some splicing factors.

Supplementary References

1. Chan, K. *et al.* An APOBEC3A hypermutation signature is distinguishable from the signature of background mutagenesis by APOBEC3B in human cancers. *Nat Genet* **47**, 1067-72 (2015).
2. Paz, I., Akerman, M., Dror, I., Kosti, I. & Mandel-Gutfreund, Y. SFmap: a web server for motif analysis and prediction of splicing factor binding sites. *Nucleic Acids Res* **38**, W281-5 (2010).
3. Piva, F., Giulietti, M., Burini, A.B. & Principato, G. SpliceAid 2: a database of human splicing factors expression data and RNA target motifs. *Hum Mutat* **33**, 81-5 (2012).
4. Pak, V., Heidecker, G., Pathak, V.K. & Derse, D. The role of amino-terminal sequences in cellular localization and antiviral activity of APOBEC3B. *J Virol* **85**, 8538-47 (2011).
5. Stenglein, M.D., Matsuo, H. & Harris, R.S. Two regions within the amino-terminal half of APOBEC3G cooperate to determine cytoplasmic localization. *J Virol* **82**, 9591-9 (2008).
6. Roberts, S.A. & Gordenin, D.A. Hypermutation in human cancer genomes: footprints and mechanisms. *Nat Rev Cancer* **14**, 786-800 (2014).
7. Seiler, M. *et al.* Somatic Mutational Landscape of Splicing Factor Genes and Their Functional Consequences across 33 Cancer Types. *Cell Rep* **23**, 282-296 e4 (2018).
