# Supplementary material for: Targeting natural splicing plasticity of APOBEC3B restricts its expression and mutagenic activity
Source: Commun Biol. 2021 Mar 22;4:386. doi: 10.1038/s42003-021-01844-5 (PMC7985488; doi:10.1038/s42003-021-01844-5)
Supplement: Supplementary file 2 — Description of Additional Supplementary File [file 42003_2021_1844_MOESM2_ESM.pdf]

Description of Additional Supplementary File

File name: Supplementary Data 1 – 8

Description: Source data in Excel format

- **Supplementary Data 1:** Expression of APOBEC3A and APOBEC3B in RNA-seq data of TCGA tumors and normal tissues (Fig. 1, 3; Supplementary Fig. 1, 5a)
- **Supplementary Data 2:** APOBEC-signature mutations of TCGA tumors (Fig. 3, Supplementary Fig. 4)
- **Supplementary Data 3:** Quantification of alternative splicing events of APOBEC3B (A3B) as Percent Spliced-in Index (PSI) in TCGA paired tumor and normal tissues (Supplementary Fig. 5)
- **Supplementary Data 4:** APOBEC3B isoform expression in HT-1376 cells for nonsense mediated decay (NMD) experiment, with biological triplicates for each experimental condition (Fig. 2c)
- **Supplementary Data 5:** APOBEC3A and APOBEC3B analysis in UROMOL study of non-muscle-invasive bladder tumors (Fig. 3b,c)
- **Supplementary Data 6:** APOBEC3B isoform expression in bladder tumors and normal tissues (Fig. 4c)
- **Supplementary Data 7:** APOBEC3B isoform expression in HT-1376 cells for pladienolide B experiment; with biological triplicates for each experimental condition (Fig. 6d)
- **Supplementary Data 8:** mRNA expression (TPMs) of splicing factors in cell lines from Cancer Cell Line Encyclopedia (CCLE) and mini-gene based A3B exon 5 skipping in cell lines quantified by densitometry of agarose gel bands for PCR products (Supplementary Fig. 6)
