# Supplementary material for: Targeting natural splicing plasticity of APOBEC3B restricts its expression and mutagenic activity
Source: Commun Biol. 2021 Mar 22;4:386. doi: 10.1038/s42003-021-01844-5 (PMC7985488; doi:10.1038/s42003-021-01844-5)
Supplement: Supplementary file 4 — Reporting Summary [file 42003_2021_1844_MOESM4_ESM.pdf]

nature research

Corresponding author(s): Ludmila Prokunina-Olsson

Last updated by author(s): 01/30/2021

Reporting Summary

Nature Research wishes to improve the reproducibility of the work that we publish. This form provides structure for consistency and transparency in reporting. For further information on Nature Research policies, see Authors & Referees and the Editorial Policy Checklist .

Statistics

For all statistical analyses, confirm that the following items are present in the figure legend, table legend, main text, or Methods section.

n/a	Confirmed	
☐	☒	The exact sample size (*n*) for each experimental group/condition, given as a discrete number and unit of measurement
☐	☒	A statement on whether measurements were taken from distinct samples or whether the same sample was measured repeatedly
☐	☒	The statistical test(s) used AND whether they are one- or two-sided<br>*Only common tests should be described solely by name; describe more complex techniques in the Methods section.*
☐	☒	A description of all covariates tested
☐	☒	A description of any assumptions or corrections, such as tests of normality and adjustment for multiple comparisons
☐	☒	A full description of the statistical parameters including central tendency (e.g. means) or other basic estimates (e.g. regression coefficient) AND variation (e.g. standard deviation) or associated estimates of uncertainty (e.g. confidence intervals)
☐	☒	For null hypothesis testing, the test statistic (e.g. *F*, *t*, *r*) with confidence intervals, effect sizes, degrees of freedom and *P* value noted<br>*Give P values as exact values whenever suitable.*
☒	☐	For Bayesian analysis, information on the choice of priors and Markov chain Monte Carlo settings
☐	☒	For hierarchical and complex designs, identification of the appropriate level for tests and full reporting of outcomes
☐	☒	Estimates of effect sizes (e.g. Cohen's *d*, Pearson's *r*), indicating how they were calculated

Our web collection on statistics for biologists contains articles on many of the points above.

Software and code

Policy information about availability of computer code

Data collection	1. TCGA data of APOBEC-signature mutations, demographic and clinical was downloaded from Broad GDAC Firehose in October 2016, via web interface at https://gdac.broadinstitute.org/<br>2. RNAseq data of APOBEC3 genes in 11,058 TCGA samples (10,328 tumors and 730 adjacent normal tissues) as BAM slices were collected from NCI Genomics Data Commons (GDC) portal accessed in August 2019 using code available at https://docs.gdc.cancer.gov/API/Users_Guide/BAM_Slicing/<br>3. RNAseq data for cell lines included in the Cancer Cell Line Encyclopedia (CCLE) was acquired as BAM files from the GDC legacy archive (https://portal.gdc.cancer.gov/legacy-archive/search/f)
Data analysis	1. Exon-exon splicing junction analysis and percent spliced-in index estimations was performed using ASpli package (version 1.5.1: https://bioconductor.org/packages/release/bioc/vignettes/ASpli/inst/doc/ASpli.pdf) on R platform (versions 3.6.0 and above).<br>2. Integrative Genomics Viewer (http://www.broadinstitute.org/igv) was used for RNAseq visualization<br>3. Clustal Omega (http://www.ebi.ac.uk/Tools/msa/clustalo/) for multiple sequence alignment of protein and nucleotide sequences<br>4. For identification of splicing regulatory elements, we used: SpliceAid 2 (http://193.206.120.249/splicing_tissue.html); SFmap (http://sfmap.technion.ac.il/); Human Splicing Finder (www.umd.be/HSF3/)<br>5. Statistical analysis was performed using generic R tools and Prism - GraphPad (version 7)

For manuscripts utilizing custom algorithms or software that are central to the research but not yet described in published literature, software must be made available to editors/reviewers. We strongly encourage code deposition in a community repository (e.g. GitHub). See the Nature Research guidelines for submitting code & software for further information.

Data

Policy information about availability of data

All manuscripts must include a data availability statement. This statement should provide the following information, where applicable:

- Accession codes, unique identifiers, or web links for publicly available datasets
- A list of figures that have associated raw data
- A description of any restrictions on data availability

The lab generated RNAseq data is available at NCBI Short Read Archive with accession number PRJNA512015T

Field-specific reporting

Please select the one below that is the best fit for your research. If you are not sure, read the appropriate sections before making your selection.

- [x] Life sciences
- [ ] Behavioural & social sciences
- [ ] Ecological, evolutionary & environmental sciences

For a reference copy of the document with all sections, see nature.com/documents/nr-reporting-summary-flat.pdf

Life sciences study design

All studies must disclose on these points even when the disclosure is negative.

Sample size	Most of the data used in this analysis is from The Cancer Genome Atlas (TCGA) and bladder cancer UROMOL study. Qualty controlled filtered data was accessed from these databases. For different types of analysis, all samples that had available matching variables required for any specific analysis were included in the analysis after removing any duplicated samples. For all cancers sufficient samples sizes were available. More specific details are provided for each analysis in the manuscript. UROMOL dataset of 460 tumors was accessed from European Genome-Phenome Archive under accession number EGAS00001001236, and an expression matrix is available under accession number ArrayExpress: E-MTAB-4321.
Data exclusions	Except duplicates no samples were excluded from the analysis.
Replication	Yes all experimental findings were reliably reproduced.
Randomization	*Describe how samples/organisms/participants were allocated into experimental groups. If allocation was not random, describe how covariates were controlled OR if this is not relevant to your study, explain why.*
Blinding	Blinding was used during data collection and analysis

Reporting for specific materials, systems and methods

We require information from authors about some types of materials, experimental systems and methods used in many studies. Here, indicate whether each material, system or method listed is relevant to your study. If you are not sure if a list item applies to your research, read the appropriate section before selecting a response.

Materials & experimental systems

n/a	Involved in the study	
☐	☒	Antibodies
☐	☒	Eukaryotic cell lines
☒	☐	Palaeontology
☒	☐	Animals and other organisms
☐	☒	Human research participants
☐	☒	Clinical data

Methods

n/a	Involved in the study	
☒	☐	ChIP-seq
☒	☐	Flow cytometry
☒	☐	MRI-based neuroimaging

Antibodies

Antibodies used

Ab, ID, Company, Dilution
1. Anti-APOBEC3A, orb19763, Biorbyt, 1:500
2. Anti-APOBEC3A, HPA043237, Millipore Sigma, 1:500
3. Anti-APOBEC3B, ab191695 Abcam 1:500
4. Anti-APOBEC3B, ab184990 Abcam 1: 500
5. Anti-DDK, MAB3118, Millipore Sigma, 1:1000
6. Anti-SF3B1, ab172634, Abcam, 1:1000
7. GAPDH, ab9485, Abcam, 1:1000

8. Anti-α-tubulin T9026 Sigma 1:10,000 Detects α-tubulin
9. Mouse-IgGκ BP-HRP, sc-516102, Santa Cruz Biotechnology, 1:5000,
10. Anti-HA 3724 Cell signaling 1:5000 Detects HA tag
11. Anti-HIV-1 p24 6458 Through NIH AIDS Reagent Program; from Dr. Michael Malim 1:10,000 Detects HIV-1 p24 protein

Secondary antibodies:

12. Anti-goat IgG-HRP, sc-2304, Santa Cruz Biotechnology, 1:5000
13. Anti-rabbit IgG-HRP, 7074, Cell Signaling, 1:5000
14. IRDye 800CW-labeled goat anti-rabbit 926-32211 LI-COR,1:10,000
15. IRDye 680-labeled goat anti-mouse 926-68070, LI-COR, 1:10,000

Validation	Data sheets were available which showed validation of these antibodies. We independently verified them for specificity as described in the manuscript

Eukaryotic cell lines

Policy information about cell lines

Cell line source(s)	All cell lines used were purchased from the American Type Culture Collection (ATCC) except pancreatic cancer cell line PATU8998T which was purchased from Leibniz Institute DSMZ-German Collection of Microorganisms and Cell Cultures (DSMZ Scientific).
Authentication	By genotyping of a panel of microsatellite markers through CGR/DCEG/NCI
Mycoplasma contamination	All cell lines in the laboratory are regularly tested for mycoplasma contamination using the MycoAlert Mycoplasma Detection kit (Lonza).
Commonly misidentified lines (See ICLAC register)	No commonly misidentified cell lines were used.

Human research participants

Policy information about studies involving human research participants

Population characteristics	Data used in this study is from TCGA and UROMOL study on human participants and has been previously described.
Recruitment	na
Ethics oversight	na

Note that full information on the approval of the study protocol must also be provided in the manuscript.

Clinical data

Policy information about clinical studies

All manuscripts should comply with the ICMJE guidelines for publication of clinical research and a completed CONSORT checklist must be included with all submissions.

Clinical trial registration	na
Study protocol	na
Data collection	na
Outcomes	na
